# Interventions to improve gross motor performance in children with neurodevelopmental disorders: a meta-analysis

**DOI:** 10.1186/s12887-016-0731-6

**Published:** 2016-11-29

**Authors:** Barbara R. Lucas, Elizabeth J. Elliott, Sarah Coggan, Rafael Z. Pinto, Tracy Jirikowic, Sarah Westcott McCoy, Jane Latimer

**Affiliations:** 1 Discipline of Paediatrics and Child Health, The University of Sydney, The Children’s Hospital at Westmead, Clinical School, Locked Bag 4001, Westmead, Sydney, NSW 2145 Australia; 2 The George Institute for Global Health, Sydney Medical School, University of Sydney, PO Box M201, , Missenden Rd, Sydney, NSW 2050 Australia; 3 Poche Centre for Indigenous Health, Sydney School of Public Health, The University of Sydney, Sydney, NSW 2006 Australia; 4 Physiotherapy Department, Royal North Shore Hospital, St Leonards, Sydney, NSW 2065 Australia; 5 The Sydney Children’s Hospital Networks (Westmead), Locked Bag 4001, Westmead, Sydney, NSW 2145 Australia; 6 School of Public Health, Curtin University, GPO Box U1987, Perth, WA 6845 Australia; 7 Pain Management Research Institute, University of Sydney at Royal North Shore Hospital, St Leonards, Sydney, NSW 2065 Australia; 8 Departamento de Fisioterapia, Faculdade de Ciências e Tecnologia, UNESP—Univ Estadual Paulista, Presidente Prudente, SP 19060-900 Brazil; 9 Division of Occupational Therapy, Department of Rehabilitation Medicine, University of Washington, Seattle, WA 98195 USA; 10 Division of Physical Therapy, Department of Rehabilitation Medicine, University of Washington, Seattle, WA 98195 USA

**Keywords:** Neurodevelopmental disorders, Motor skills disorders, Motor skills, Child development, Physiotherapy, Cerebral palsy, Developmental Coordination Disorder

## Abstract

**Background:**

Gross motor skills are fundamental to childhood development. The effectiveness of current physical therapy options for children with mild to moderate gross motor disorders is unknown. The aim of this study was to systematically review the literature to investigate the effectiveness of conservative interventions to improve gross motor performance in children with a range of neurodevelopmental disorders.

**Methods:**

A systematic review with meta-analysis was conducted. MEDLINE, EMBASE, AMED, CINAHL, PsycINFO, PEDro, Cochrane Collaboration, Google Scholar databases and clinical trial registries were searched. Published randomised controlled trials including children 3 to ≤18 years with (i) Developmental Coordination Disorder (DCD) or Cerebral Palsy (CP) (Gross Motor Function Classification System Level 1) or Developmental Delay or Minimal Acquired Brain Injury or Prematurity (<30 weeks gestational age) or Fetal Alcohol Spectrum Disorders; and (ii) receiving non-pharmacological or non-surgical interventions from a health professional and (iii) gross motor outcomes obtained using a standardised assessment tool. Meta-analysis was performed to determine the pooled effect of intervention on gross motor function. Methodological quality and strength of meta-analysis recommendations were evaluated using PEDro and the GRADE approach respectively.

**Results:**

Of 2513 papers, 9 met inclusion criteria including children with CP (*n* = 2) or DCD (*n* = 7) receiving 11 different interventions. Only two of 9 trials showed an effect for treatment. Using the *least conservative* trial outcomes a large beneficial effect of intervention was shown (SMD:-0.8; 95% CI:-1.1 to −0.5) with “very low quality” GRADE ratings. Using the *most conservative* trial outcomes there is no treatment effect (SMD:-0.1; 95% CI:-0.3 to 0.2) with “low quality” GRADE ratings. Study limitations included the small number and poor quality of the available trials.

**Conclusion:**

Although we found that some interventions with a task-orientated framework can improve gross motor outcomes in children with DCD or CP, these findings are limited by the very low quality of the available evidence. High quality intervention trials are urgently needed.

**Electronic supplementary material:**

The online version of this article (doi:10.1186/s12887-016-0731-6) contains supplementary material, which is available to authorized users.

## Background

Development of motor function is important for skill acquisition, and enabling children to participate fully in school and leisure activities [1]. It is also important for establishing lifelong, physical activity patterns for healthy development into adulthood [2]. Gross motor skills use large muscle groups for coordinated body movements such as walking, running, jumping and the maintenance of balance. They are fundamental to childhood development as they underpin functional activities, play and social interaction and in older aged children support complex movement skills required for sport and fitness [3]. School age studies show that gross motor skills are integral to social, recreational and academic participation and have been linked to healthy self-esteem [4] and cognitive development [5]. Furthermore, poor gross motor performance may incline children towards activity avoidance and sedentary behaviors linked to an increased risk of chronic disease in adulthood [6–8].

Poor gross motor function may be caused by a range of neurodevelopmental disorders such as developmental co-ordination disorder (DCD), cerebral palsy (CP) diplegia, developmental delay (DD) or minimal acquired brain injuries [9] which result in mild to moderate gross motor deficits in children. Physiotherapists are frequently asked to assess such children and advise on their management.

Intervention strategies for children with gross motor disorders need to address specific skill deficits as well as provide opportunities for regular fun physical activity. The latter may be vital to establishing long term healthy fitness habits for adulthood [2]. Interventions may be described as *traditional*: a combination of a variety of sensory integrative, gross motor, fine motor and perceptual-motor activities [10]; *process-orientated*: specifically designed kinaesthetic tasks; or *task-orientated strategies*: practicing real life activities with the intention of acquiring skill [11]. Importantly, clinicians should base treatment decisions on evidence, using interventions most likely to produce the greatest improvement in motor outcomes. Whilst several theoretical models of motor learning influence treatment design [12], researchers are now seeking how best to optimize motor learning and harness the continuous spontaneous plasticity of the brain in early childhood through specific therapy content [13]. Models of neuroplasticity [14, 15] indicate the benefits of early detection and the use of interventions to ameliorate gross motor disorders in early childhood. Key components of successful therapy content are still unclear. Promising results using enriched environments [16] and complex motor training [17, 18] to optimize neuroplasticity of damaged neural structures have already been demonstrated in rats with prenatal alcohol exposure (PAE). Similarly, goal-oriented, activity-based, environmental enrichment therapy to optimize neuroplasticity and brain injury recovery is being explored to improve motor outcomes in infants at high risk of cerebral palsy [13, 19]. Further work is needed to translate these encouraging developments in therapy content into evidenced-based mainstream clinical practice.

The effectiveness of current therapy or treatment options for these children with mild to moderate gross motor disorders is unknown. Common neurodevelopmental conditions considered within the mild to moderate range as determined by expert peer consensus include children with Developmental Co-ordination Disorder (DCD) [10, 20] Cerebral Palsy (CP) classified as Gross Motor Function Classification System Level I (GMFCS I) [21, 22], Developmental Delay or gross motor delay (one SD or more, ≤16th centile) below the standardised population mean [23], minimal acquired brain damage (Glasgow Coma Score ≥ 13) [24, 25], children with a history of extreme prematurity (≤29 weeks gestational age) [26] and extremely low birth weight (<1000 g birth weight) [27] and fetal alcohol spectrum disorder (FASD) [28]. Despite the commonality of referral in physiotherapy clinical practice for management of these children, there are no previously published rigorous systematic reviews. This systematic review arose following an urgent need to find evidence-based treatments for children presenting with mild to moderate gross motor disorders. We aimed to identify relevant trials and determine the effectiveness of conservative interventions (i.e. non-surgical, non-pharmacological) compared to no treatment or usual care on gross motor performance in children aged 3 to ≤18 years with mild to moderate neurodevelopmental pediatric disorders where similar gross motor delay occurs.

## Methods

### Design

A systematic review of intervention studies with meta-analysis was conducted guided by the Preferred Reporting Items for Systematic reviews and Meta-Analyses (PRISMA) statement [29]. National and international expert clinicians and researchers were contacted to determine the range of conditions considered to present with mild to moderate gross motor disorders. The study protocol was prospectively registered with PROSPERO (register number CRD42014009493, web link: http://www.crd.york.ac.uk/prospero/display_record.asp?ID=CRD42014009493.

### Data sources and searches

Electronic data sources were systematically searched from January 1980 to June 8, 2015 using a highly sensitive search strategy (Additional file 1). Data sources included: MEDLINE, EMBASE, AMED, CINAHL, PsycINFO, The Cochrane Collaboration, PEDro and Google Scholar. Additional references were found by hand searching reference lists of relevant studies, conference abstracts, registered clinical trials (Australia and New Zealand Clinical Trials Registry, and the World Health Organization; International Clinical Trials Registry Platform) and by approaching experts in the field. Searches were restricted by language to English publications.

### Study selection

Two reviewers (BL and SC) screened all relevant titles and abstracts of the retrieved publications to exclude irrelevant titles. They independently assessed the full reports for eligibility against the inclusion and exclusion criteria (Table 1) using standardized forms (Additional file 2).

**Table 1:** Inclusion and exclusion criteria

Inclusion criteria	
Design	
Human intervention studies including randomized controlled trials, quasi randomized controlled trials and randomized cross-over trials.	
Participants	
Aged between 3 to ≤ 18 years.	
Conditions	
Fetal Alcohol Spectrum Disorders (FASD) diagnoses determined using internationally recognised standardised diagnostic criteria.	
Developmental Co-ordination Disorder (DCD) determined using internationally recognised diagnostic criteria such as the DSM 4 or 5.	
Cerebral Palsy (CP) classified at Gross Motor Function Classification System Level I.	
Extremely preterm or extremely low birth weight children born at ≤ 30 weeks gestational age, < 1000 g with mild – moderate GM disorders.	
Acquired Minimal Brain Injury or mild Traumatic Brain Injury (Glasgow Coma Score ≥ 13).	
Developmental Delay determined using internationally recognised standardised diagnostic criteria defined by the DSM 4 or 5 in children ≤ 5 years age.	
Gross motor delay including children functioning at 1SD (16^th^ centile) below the standardised population mean assessed by a standardised assessment tool.	
Interventions	
Any home, community or school-based non-pharmacological, non-surgical intervention for children and adolescents involving a targeted therapy with stated clear intent to improve gross motor proficiency delivered by a trained health professional (e.g. Physiotherapist, Occupational Therapist).	
Comparator (s)/control	
No treatment, placebo, waiting list or usual therapy	
Primary Outcomes	
GM performance measured with a standardised assessment tool.	
Secondary Outcomes	
Compliance, parental satisfaction, child satisfaction and cost.	
Exclusion Criteria	
Exclusion Criteria	
Studies not reporting a quantitative effect size including either a standard error (SE), standard deviation (SD) or confidence interval (CI).	
Studies including subjects with:	
Chromosomal disorders known to be associated with a motor deficit.	
Unadjusted hearing or visual impediments.	
Moderate to severe intellectual disability with IQ below 60	
Dystonia or hip dysplasia	
Studies reporting non-conservative rehabilitation interventions including surgery and pharmacological management (e.g. Botox therapy, dorsal rhizotomy).	

### Data extraction and quality assessment

The same reviewers (BL and SC) independently extracted data using standardized forms (Additional file 3). Disagreements were resolved by discussion with other authors (JL and RP). From studies meeting eligibility criteria, information was extracted on condition, age, study design, intervention, comparator control and statistical analysis including means (final scores or change score), standard errors (SE) or standard deviations (SD) or confidence interval (CI), and sample sizes. The primary outcomes were gross motor performance measures such as ball skills, balance, co-ordination evaluated with a standardised assessment tool (Table 1). The secondary outcomes were compliance, parental satisfaction, child satisfaction and cost. Studies required measurement of a gross motor outcome using a standardized assessment tool (Table 2).

**Table 2:** Assessment of quality using PEDro item criterion

Internal Validity	
Random allocation.	
Concealed allocation.	
Similarity of baseline on key measures.	
Subject blinding.	
Therapist blinding.	
Assessor blinding.	
> 85% follow-up of at least one outcome.	
Intention- to- treat analysis.	
Interpretability	
Between-group statistical comparison for at least 1 key outcome.	
Point estimates and measures of variability provided by at least 1 key outcome.	

Each of criterions was explicitly judged using: 1 = present or 0 = absent. A quality score (maximum score = 10) was allocated to each individual study. Eligibility criteria and source of participants were also assessed as part of the PEDro scale criterion but were not included in the quality score as per the PEDro scoring system

Two trained independent raters assessed the studies’ quality (BL and SC) using the 10-point Physiotherapy Evidence Database (PEDro) scale which has established validity and reliability [30, 31]. This checklist is designed to rate clinical trials on internal validity and sufficient statistical information to ensure interpretability [32]. If trials were already listed on the PEDro database we adopted these scores. Disagreements were arbitrated by a third reviewer (JL). Table 2 shows the criteria for quality assessment.

The Grading of Recommendations Assessment Development and Evaluation (GRADE) approach was used to evaluate the overall quality of evidence and to indicate the strength of meta-analysis recommendations based on the evidence [33]. A modified version was used where the GRADE classification was downgraded by 1 level for each of the 5 factors we considered: 1. Design limitations (25% or more of trials had poor study design defined by PEDro score < 7), 2. Inconsistent results (25% or more of the trials have results that are not in the same direction), 3. Imprecision (<300 participants for each outcome), 4. Indirectness (drawing conclusions about treatment effects from another population which is not PAE exposed) [33, 34] and 5. Reporting bias (a funnel plot showing evidence of small study effects) [35].

The funnel plots of the *least* and *most conservative* gross motor outcome standardized mean difference (SMD) were assessed for small study effects. These were visually inspected for asymmetry and quantified using the Egger test [36, 37]. If the Egger test was statistically significant (2-tailed *P* < 0.100), the quality of the meta-analysis was downgraded by 1 level. Two independent raters (BL and RZP) judged whether the 5 factors were present for each outcome. The following definitions of quality of evidence were applied; *high quality*: all domains satisfied, high confidence that the true effect lies close to that of the estimate of the effect; *moderate quality*: 1 domain not met, moderate confidence in the effect estimate: the true effect is likely to be close to the estimate of the effect, but there is a possibility that it is substantially different; *low quality*: 2 domains not met, limited confidence in the effect estimate, the true effect may be substantially different from the estimate of the effect, and *very low quality*: 3 or more domains not met, little confidence in the effect estimate: the true effect is likely to be substantially different from the estimate of effect [38].

### Data synthesis and analysis

The primary outcomes were gross motor performance measures derived using a standardized assessment tool. Mean scores, SD or SE for continuous measures of gross motor performance, and sample size from each group (intervention and comparator) were used to calculate the standardized mean difference (SMD) and 95% confidence intervals (CI). Where separate gross motor outcomes associated with a motor composite score were not reported, the authors were contacted to provide this. If trials reported outcomes as graphs, the mean scores and standard deviations were estimated from these graphs. Where trials included subjects with a variety of conditions, only data pertaining to conditions of interest were extracted. If the trial was a multiple-arm RCT, all relevant experimental intervention groups and the control group had data extracted. In follow-up studies with multiple time points, only data closest to the end of the intervention were included. In randomized cross-over trials, size effects were only extracted at the first cross-over point to avoid contamination with subsequent intervention regimes.

Due to the small number of trials that reported a number of different interventions it was not possible to meaningfully group studies according to intervention for the meta-analyses. Therefore the meta-analyses were conducted as follows. Firstly a meta-analysis that used the gross motor outcome measure with the *most conservative* SMD from each trial was performed. A second meta-analysis that used the gross motor outcome measure with the *least conservative* SMD from each trial was then performed. An overall pooled effect was calculated for each meta-analysis where the magnitude of the SMD was interpreted as follows: small, SMD = 0.2; medium, SMD = 0.5; and large, SMD = 0.8 [39]. For trials with only one gross motor outcome, the same outcome measure was entered into both meta-analyses. Where two studies reported data from the same cohort, only one study was included in the meta-analysis. If more than one comparison from a multiple-arm RCT was included in the meta-analysis, the control group sample size was divided by the number of relevant intervention groups according to Cochrane guidelines [40, 41]. Forest plots were used to visually assess the SMD and 95% CI of each study and funnel plots to assess for publication bias and small study effects.

Analyses were performed using the Comprehensive Meta-Analysis software, version 2.2.04 (Biostat Eaglewood, NJ) using a random effects model [42]. Statistical significance was set at 0.05 and heterogeneity was analyzed using the I^2^ statistic. Trials were considered sufficiently homogeneous for meta-analyses pooling with *I* ^2^ ≤ 25% [40, 42]. The outcomes of studies not reported in the meta-analysis were described individually.

## Results

### Literature search

**Fig. 1:**
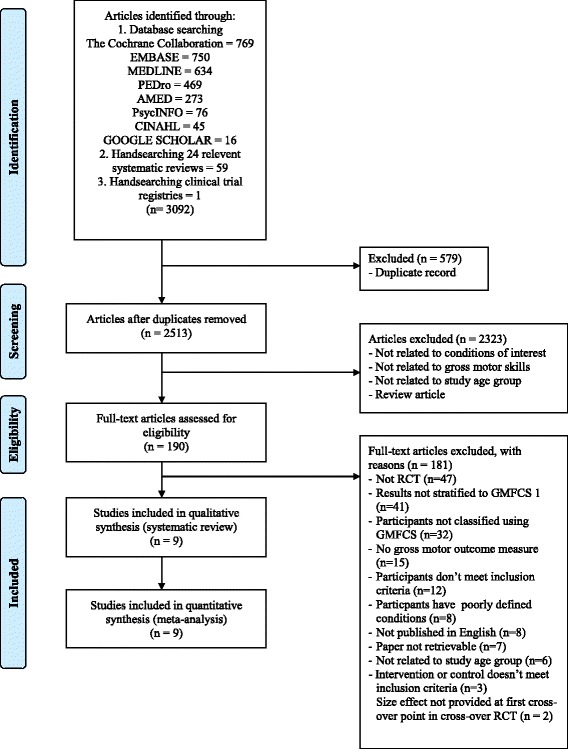
Identification and selection of studies for the review

The data base searches identified 3032 studies and hand searching from systematic reviews and clinical trials registries retrieved 60 more papers (3092 total). No additional studies were found from other sources. After duplicates were removed and citations were screened by title and abstract, 190 eligible articles were considered for inclusion by reviewing full articles, using exclusion criteria listed in Fig. 1. The systematic review included a total of nine published intervention trials [10, 43–50], all of which were included for consideration in the meta–analysis for the least and most conservative forest plots (Fig. 1).

### Characteristics of included trials

The nine included trials were RCT’s (8/9) [10, 43–49] or cross-over RCT’s (1/9) [50] with two trials derived from the same cohort [48, 49]. Participants included children diagnosed with CP GMFCS I (*n* = 2) [44, 47] or DCD (*n* = 7). No trials of other conditions were found that met inclusion criterion. Participants’ age ranged from 5 to 18 years. Where gender was reported, not unexpectedly in the DCD RCT’s (6/7) the majority of participants were male (male: female ratio; 5:2) this being a feature of DCD diagnosis [51] . In the one CP trial gender was equally distributed (1/2). Two trials authors [45, 46] were contacted to retrieve gross motor data from their data files to enable inclusion in the meta-analysis.

**Fig. 2:**
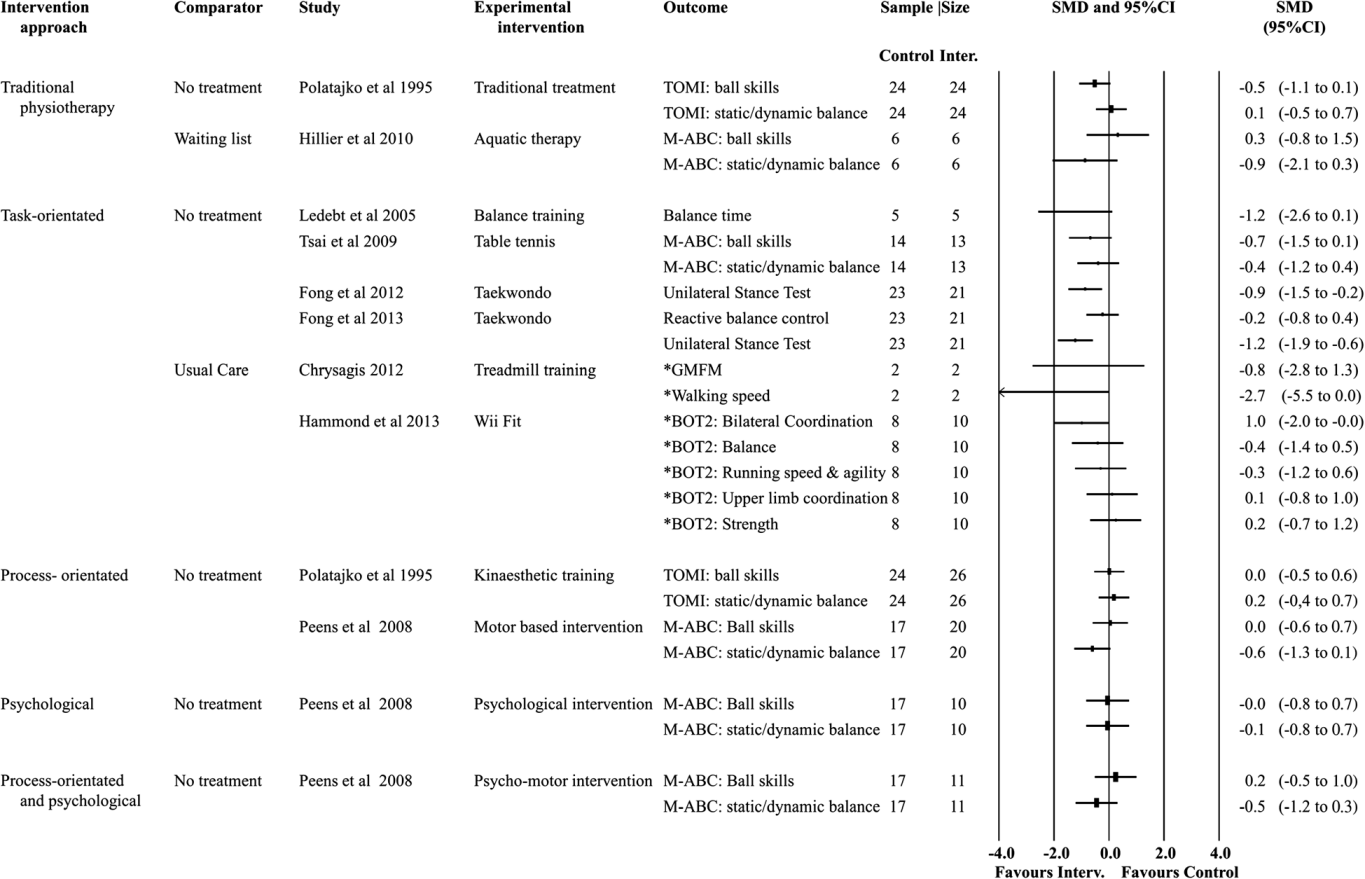
Forest plot—all treatment effects

The nine trials reported 11 different experimental interventions. In children with a diagnosis of CP GMFCS I these included treadmill training without body weight support [47] and balance training with visual feedback [44]. In children with a diagnosis of DCD these included Taekwondo [48, 49], aquatic therapy [46], table tennis [45], a commercially available home video game console “Wii Fit” [50],“psychological” intervention [42], “psychomotor” intervention [43], “motor” intervention [43], process orientated [10] and traditional intervention (any combination of a variety of sensory integrative, gross motor, fine motor and perceptual-motor activities to meet the specific motor needs of the child) [10]. Two trials of the same cohort reported a Home Exercise Program (HEP) (2/9) as part of their intervention strategy to improve gross motor skill. It involved daily practice of activities performed during training except on formal training days with monitoring by a log book [48, 49]. The trials were grouped according to intervention and comparator type (Fig. 2). Comparator “interventions” categorised as *usual care* (2/9) included “treatment as usual” [50] and “conventional physiotherapy” [47]; categorised as *waiting list* (1/9) included only “wait list” [46]; and categorised as *no treatment* (6/9) included “no treatment” [50] and “no training” [43–45, 48, 49].

All trials performed interventions over multiple sessions reporting that the programs were delivered over 4 to12 weeks (mean ± SD: 8.3 ± 32.8), at a frequency of 1 to 3 times/week (mean ± SD: 1.9 ± 1.0), with sessions lasting 10 to 60 mins (mean ± SD: 38.9 ± 17.5). Most trials used standardised assessment tools (8/9) to measure gross motor outcome with six different tools employed: the Movement Assessment Battery for Children (3/11) [43, 45, 46], Unilateral Stance Test (2/11) [48, 49], Motor Control Test (2/11) [48, 49], Gross Motor Function Measure (1/11) [47], Bruininks Oseretsky Test of Motor Proficiency – Second Edition (1/11) [50], and Test of Motor Impairment (1/11) [10]. Two trials used other standardised measures; walking speed [47] and % time balancing on a target [44]. All studies provided post-test measures after treatment cessation but only 3 studies included longer term follow-up at 8 weeks [43], 10 weeks [44] and 14 weeks [50]. Further information about study characteristics is described in Table 3.

**Table 3:** Systematic review: Individual study characteristics (*n* = 9)

Reference	Study design	Details of participants	Intervention	Intervention dose regimens	Outcomes (measures)	Intervention approach	PEDro score
Polatajko et al. 1995 [10]	Randomised control trial	Source: children referred to the Home Care School Program Middlesex, UK Age: 7 to 13 years. Diagnosis: DCD *N* = 74 Gender (male/female): not reported	Group 1 Kinaesthetic training vs no treatment Group 2 “Traditional treatment” vs no treatment	Intervention Group 1 (*n* =26) Two to three 20 min sessions per week for a maximum of 12 sessions over 5 weeks or until the child could perform the task to criteria. Group 2 (*n* = 24) Two to three 45 min sessions per week for a total of 24 sessions over 9 weeks involving sensory integrative, gross motor, fine motor and perceptual motor interventions Control (*n* = 26) No treatment	Primary Gross motor skills (TOMI; Ball skills; static and dynamic balance Secondary None reported Post-test measures Group 1: 13 weeks Group 2: 13 weeks Group 3: 9 weeks after the end of treatment	Group 1 Process- orientated Group 2 Traditional	6
Ledebt et al. 2005 [44]	Randomised control trial	Source: medical centre of Vrije Universiteit, Amsterdam Age: 5 to 11 years Diagnosis: CP GMFCS1 - spastic hemiplegia *N* = 10 Gender: not reported.	Balance training (to improve gait) vs no training	Intervention (*n* = 5) 18 sessions total; three 30 min sessions per week for 6 weeks; static and dynamic balance tasks included. Control (*n* = 5) No training	Primary Balance (Centre of Pressure force platform measures during quiet and dynamic stance balance) Gait (step length symmetry in gait) Secondary None reported Post-test measures Time 1: 6–7 weeks post baseline Time 2: no later than 10 weeks post time 1	Task-orientated	3
Peens et al. 2008 [43]	Randomised control trial	Source: nine different primary schools in the Potchesfstroom district in North-west Province of South Africa Age: 7 to 9 years Diagnosis: DCD *N* = 58 Gender (male/female): not reported	Group 1 “Motor based” intervention vs no intervention Group 2 Psychological intervention vs no intervention Group 3 Psycho-motor intervention vs no intervention	Intervention Group 1 (*n* = 20) Two 30 min sessions per week for 8 weeks involving task specific kinaesthetic and sensory integration interventions Group 2 (*n* = 10) Weekly 45 mins intervention for 8 weeks involving self-concept enhancement Group 3 (n = 11) Three sessions per week for 8 weeks involving two 30 min “motor” based sessions and one 45 min psychological session (as described above) Control (*n* = 17) No intervention	Primary Gross motor skills (TOMI; Ball skills; static and dynamic balance) Secondary Child self-concept (TSCS –CF) Anxiety (CAS) Post-test measures All groups at 8 and 16 weeks	Group 1 Process- orientated Group 2 Psychological Group 3 Process- orientated and psychological	4
Tsai et al. 2009 [45]	Randomised control trial	Source: mainstream classrooms in southern Taiwan Age: 9 to 10 years Diagnosis: DCD *N* = 27 Gender (male/female): not reported	Table tennis vs regular class room activities and no training	Intervention (*n* = 13): Three 50 min training sessions per week over a 10 week period. Training intervention performed in sequence of increasing complexity. Control (*n* =14): No treatment	Primary Gross motor skills (M-ABC; Ball skills and Static/dynamic balance categories) Secondary None reported Post-test measures At 10 weeks	Task-orientated	3
Hillier et al. 2010 [46]	Randomised control trial	Source: Minimal Motor Disorder Unit of Women’s and Children’s Hospital, Adelaide, Australia Age: 5 to 8 years Diagnosis: DCD *N* = 13 Gender (male/female): not reported	Aquatic therapy vs waiting list	Intervention (n = 6) Weekly 30 min sessions over a 6–8 week period (maximum of 6 sessions) in 1:1 format involving task specific training of ball skills, standing balance and walking/running. Control (*n* = 6) Waiting list.	Primary Gross motor skills (M-ABC; Ball skills and Static/dynamic balance categories) Secondary Child’s self-concept (PSPCSA) Parent’s perception of changes in their child’s participation (0–5 Likert scale) Post-test measures End of the 6th session ie 6–8 weeks	Traditional	7
Chrysagis et al. 2012 [47]	Randomised control trial	Source: special school for students with physical disabilities, Athens, Greece Age: 15 to 18 years Diagnosis: CP GMFCS1 - spastic diplegia *N* = 4 Gender (male/female): 0/4	Treadmill training without body weight vs individual gross motor activities (conventional physiotherapy).	Intervention (*n* = 2) Three 30 min sessions per week over 12 weeks. Each session included a 10 min warm-up and 5 min cool-down Control (*n* = 2) Three 45 min sessions per week over 12 weeks. Each session consisted of three 15 min sets of mat activities, balance and gait training and functional gross motor activities (i.e. usual care)	Primary Gross motor function (GMFM) Gait (self-selected walkingspeed) Secondary None reported Post-test measures End of 12 weeks	Task-orientated	8
Fong et al. 2012 [48]	Randomised control trial	Source: local child assessment centres and hospitals, Hong Kong Age: 6 to 9 years. Diagnosis: DCD *N* = 44 Gender (male/female): 35/9 Intervention group includes Asperger syndrome (*n* = 2), Autistic spectrum disorder (*n* = 1) Control group includes Asperger syndrome (*n* = 3)	Taekwondo vs no training	Intervention (*n* = 21) Weekly 1 h session of training for 12 consecutive weeks (including daily home exercise program) Control (*n* = 23) No training	Primary Static balance (Unilateral Stance Test using non-dominant leg) Sensory organisation of balance (Sensory Organisation Test) Secondary Compliance to daily home exercise program monitored by log book (based on activities from Taekwondo sessions) Post-test measures End of 12 weeks	Task-orientated	6
Fong et al. 2013 [49]	Randomised control trial	Source: local child assessment centres and hospitals, Hong Kong Age: 6 to 9 years. Diagnosis: DCD *N* = 44 Gender (male/female): 35/9 Intervention group includes Asperger syndrome (*n* = 2), Autistic spectrum disorder (*n* = 1) Control group includes Asperger syndrome (*n* = 3)	Taekwondo vs no training	Intervention (*n* = 21) Weekly 1 h session of training for 12 consecutive weeks (including daily home exercise program) Control (*n* = 23) No training	Primary Static balance (Unilateral Stance Test using dominant leg) Reactive balance (Motor Control Test) Muscle strength (isokinetic concentric knee flexion and extension) Secondary Compliance to daily home exercise program monitored by log book (based on activities from Taekwondo sessions) Post-test measures End of 12 weeks	Task-orientated	6
Hammond et al. 2014 [50]	Randomised crossover controlled trial	Source: two primary schools in Mid-Sussex, UK Age: 7 to 10 years Diagnosis: DCD *N* = 18 Gender (male/female): 14/4	Wii Fit vs usual care Phase 1 Wii Fit vs usual care Phase 2 Usual care vs Wii Fit 2.5 months between Phase 1 and 2	Intervention (*n* = 10) Weekly 10 mins of supervised play 3 times over a 4 week period. Children could choose from 8 Wii – Fit games which focus on balance and coordination. Control (*n* = 8) Usual care: 1 h per week of school-run Jump Ahead intervention practicing “motor skills”	Primary Gross motor skills (BOT-2 SF; bilateral-coordination, strength, balance, running speed and agility, upper limb co-ordination) Secondary Child satisfaction (CSQ) Post-test measures Phase 1: End of week 4 Phase 2: End of week 18	Task-orientated	5

*BOT-2 SF*: Bruininks Oseretsky Test of Motor Proficiency – Second Edition, Short Form, *CAS*: Child Anxiety Scale, *CSQ*: The Co-ordination Skills Questionnaire, *DCD*: Developmental Coordination Disorder, *FES*: Functional Electrical Stimulation, *GMFCS 1*: Gross Motor Function Classification System Level 1, *GMFM*: Gross Motor Function Measure, *M-ABC*: Movement Assessment for Children, *MCT*: Motor Control Test, *PSPCSA*: Pictorial Scale of Perceived Competence and Social Acceptance, *SOT*: Sensory Organisation Test, *TSCS-CF*: The Tennessee Self-Concept Scale (Child Form), *TOMI*: Test of Motor Impairment, *UST*: Unilateral Stance Test, *UK*: United Kingdom, *USA*: United States of America

### Risk of bias and assessment of quality

The methodological quality assessment using the PEDro scale (a score out of 10—Table 4) showed a mean score of 5.3 (SD: 1.7) suggesting moderate quality [52]. The majority of studies (7/9) scored ≤ 6, hence using GRADE criteria both meta-analyses had their quality of evidence downgraded because of design limitations. The most common methodological flaw was omission of blinding. Whilst it is acknowledged that it may not be possible to blind therapists (0/9) or subjects to the intervention (1/9), less than half of the included studies had blinded assessors (4/9) measuring post intervention outcomes. Intention to treat analysis was used in few studies (3/9). Visual inspection of both funnel plots (Additional files 4 and 5) found relative symmetry except for one outlier in the *least conservative* funnel plot possibly indicating small studies effects. The statistical significance of the statistical Egger test (*most conservative*; *p* = 0.34 (95% CI: −3.46 to 1.39), *least conservative*; *p* = 0.08 (95% CI: −3.21 to 0.25)) supports these observations. Hence the *least conservative* meta-analysis had the GRADE quality of evidence downgraded because of small study bias.

**Table 4:** Systematic review: PEDro ratings for eligible trials (*n* = 9)

Study	Random allocation	Concealed allocation	Baseline comparability	Blinding of subjects	Blinding of therapists	Blinding of assessors	Adequate follow-up	Intention to treat analysis	Between-group comparisons	Point estimates and variability	Total score/out of 10
Chrysagis 2012 [47]	+	+	+	+	–	–	+	+	+	+	8
Fong 2012 [48]	+	–	+	–	–	+	–	+	+	+	6
Fong 2013 [49]	+	–	+	–	–	+	–	+	+	+	6
Hammond 2013 [50]	+	–	+	–	–	–	+	–	+	+	5
Hillier [46]	+	+	+	–	–	+	+	–	+	+	7
Ledebt 2005 [44]	+	–	+	–	–	–	–	–	+	–	3
Peens 2008 [42]	+	–	+	–	–	–	–	–	+	+	4
Polatajko 1995 [43]	+	–	+	–	–	+	+	–	+	+	6
Tsai 2009 [45]	–	–	+	–	–	–	–	–	+	+	3

### Primary outcome measures

**Fig. 3:**
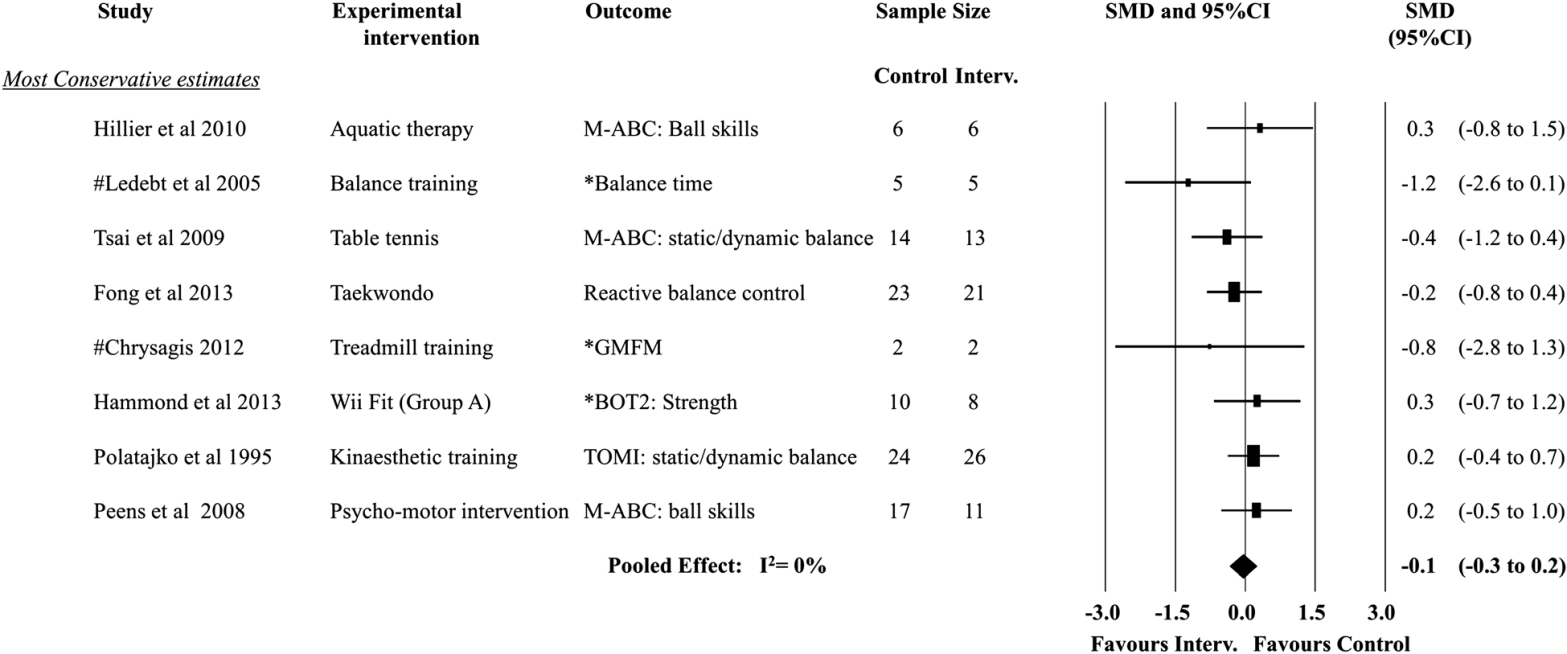
Forest plot−most conservative treatment effects

**Fig. 4:**
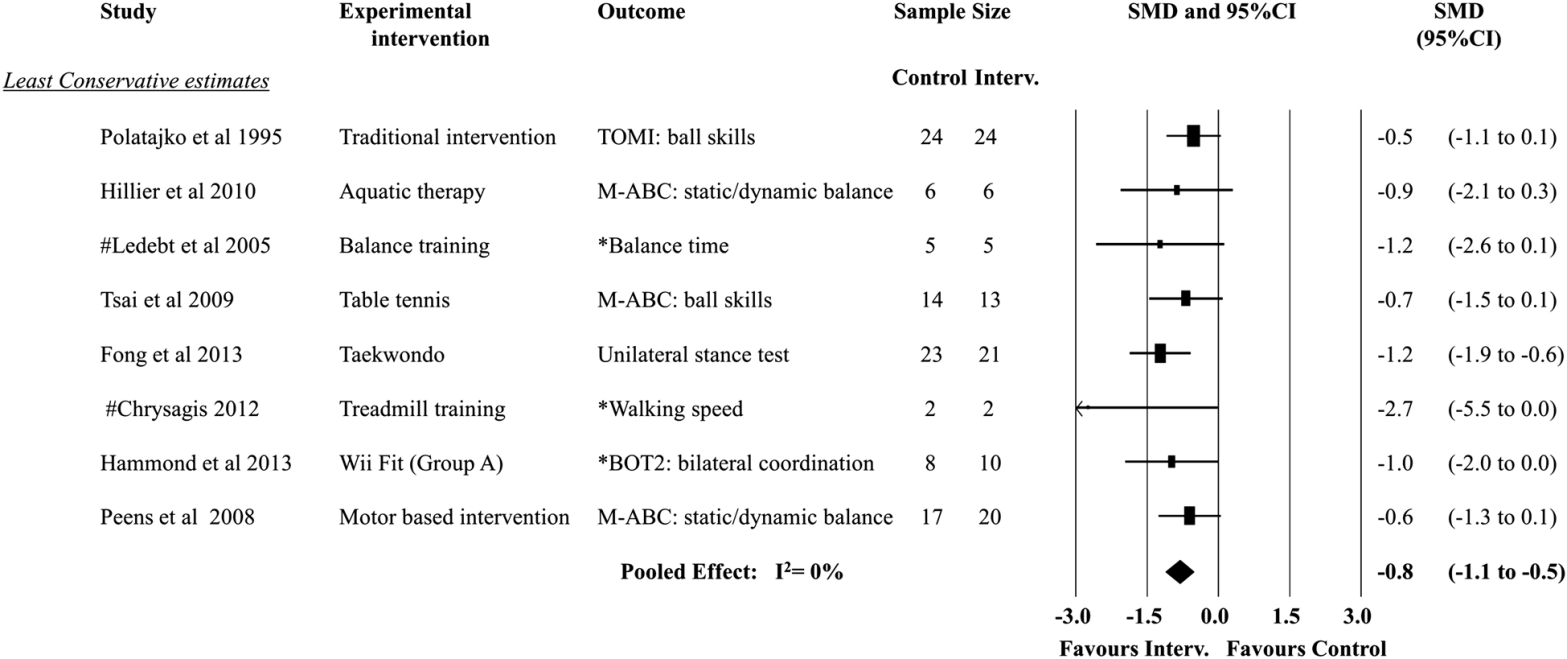
Forest plot−least conservative treatment effects

Of the nine trials included in the systematic review, one used a two-arm design [10], one a three-arm design [43] and one a crossover design [50]. Intervention effects for all studies are presented in Fig. 2. Data were available from all studies for pooling and were used in the meta-analyses for both the highest and lowest trial gross motor outcomes. Figure 3 shows the *most conservative* SMD for gross motor outcomes in each trial. The results suggest no overall pooled effect (SMD: −0.1; 95% CI:-0.3 to 0.2, random effects meta-analysis, *I* ^2^ = 0%). No trial outcome SMD was significant for the following interventions: aquatic therapy, balance training, table tennis, treadmill training, Wii Fit, Kinaesthetic Training and Psychomotor intervention. The quality of evidence (GRADE – Table 5) for this pooling was rated as “low quality” (downgraded for limitation of study design and imprecision). Figure 4 shows the *least conservative* SMD for gross motor outcomes in each trial. The results suggest a large size [39] overall pooled effect (SMD: −0.8; 95% CI:-1.1 to −0.5, random effects meta-analysis, *I* ^2^ = 0%). Only Taekwondo showed a significant treatment effect (SMD: −1.2; 95% CI: −1.9 to −0.6) with the SMD effect size interpreted as large [39]. No other trial outcome SMD was significant for the following interventions: traditional intervention aquatic therapy, balance training, table tennis, motor based intervention, treadmill training, Wii Fit and motor based interventions. The quality of evidence (GRADE – Table 5) for this pooling was rated “very low quality” (downgraded for limitation of study design, imprecision and reporting bias).

**Table 5:** Meta-analysis: Quality of outcome assessment summary

Studies	Quality assessment				Patients, *n*		Effect^a^		Quality
	Limitation of study design	Inconsistency	Imprecision	Indirectness	Reporting Bias	Intervention Group	Comparator Group	SMD^b^ (95% CI)	
Most Conservative^h^	Serious risk^c^	No serious inconsistency^d^	Serious imprecision^e^	Trial context similar^f^	Undetected^g^	159	178	−0.1 (−0.3 to −0.2)	Low quality
Least Conservative^h^	Serious risk^c^	No serious inconsistency^d^	Serious imprecision^e^	Trial context similar^f^	Detected^g^	159	178	−0.8 (−1.1 to −0.5)	Very low quality

^a^Positive values favour the intervention group

^b^The SMD of the intervention group compared to the comparator group

^c^More than 25% of the participants from studies with low methodological quality (Physiotherapy Evidence Database score < 7 points)

^d^25% of more of trials don’t have findings in the same direction

^e^Fewer than 400 participants for each outcome

^f^Trial context is not exactly the same as the review question

^g^ Inspection of funnel plot asymmetry

^h^meta-analysis studies included (*n* = 9)

### Secondary outcome measures

Studies rarely reported secondary outcome measures. No studies reported child and parent satisfaction or cost outcomes. Two studies from the same cohort reported compliance (92.5%) related to a HEP supplementing Taekwondo training [48, 49] (Table 4).

### Studies not included in the final meta-analyses pooling

Only one study [48] was not included in the *leas*t or *most conservative* meta-analysis pooling and was derived from the same cohort as another study in the meta-analyses [49]. Authors reported unilateral stance of the non-dominant leg improved in children with DCD after Taekwondo training (SMD:−0.9; 95% CI:−1.5 to−0.2). This study contained neither the least nor most conservative gross motor outcomes.

## Discussion

To our knowledge this is the first systematic review and meta-analysis to judge the effectiveness of interventions to improve gross motor performance in children with mild to moderate gross motor disorder; a common reason for referral to physiotherapy outpatient services. Current trials were conducted in Hong Kong (*n* = 2), UK (*n* = 2), Australia (*n* = 1), Greece (*n* = 1), Netherlands (*n* = 1), South Africa (*n* = 1), Taiwan (*n* = 1) and in only two conditions (CP and DCD). When the analysis is conducted using the most optimistic interpretation of the trial data (that is using the *least conservative* SMD) the pooled estimates demonstrate a large effect for conservative intervention, but the quality of the overall evidence is *very low quality* according to GRADE. The available evidence only shows favorable effects of large size in Taekwondo (*n* = 44) [49] in children with DCD. When the analysis is conducted using the least optimistic interpretation of the data (that is using the *most conservative* SMD) the pooled estimates demonstrate no effect. Therefore, while this review found some evidence that intervention can improve gross motor outcomes in children with DCD or CP, the very low quality of evidence suggests little confidence in the estimate of this effect. These data reveal that there is little high quality evidence to guide interventions for children with mild to moderate gross motor disorders.

There are a number of earlier reviews that have explored the effectiveness of interventions on motor performance in patients with traumatic brain injury [24]^,^ and DCD [11, 20, 53, 54] or associated with different types of intervention (e.g. virtual reality [55] or interactive computer play [56]). Most of these reviews have considered motor performance [11, 20, 53–56] in total rather than gross motor performance alone, with only one including RCT’s [20] alone, while two of these reviews [53, 54] were conducted over 10 years ago. Only two of these reviews used meta-analysis to estimate an overall treatment effect [20, 53] with the GRADE approach not used to appraise the quality of the evidence in either. Both reviews found that the intervention approach chosen was important, their results finding strong treatment effects for task-orientated interventions [20, 53] and traditional physical therapies or occupational therapies [53]. We would categorise the 11 interventions reported in our nine included studies into the following therapy approaches: (i) *task–orientated*: table tennis, treadmill training, Wii Fit, balance, taekwondo; *process-orientated*: process-orientated intervention; *traditional*: traditional treatment, motor based intervention; aquatic therapy; and *other*: psychological. Our review too found some evidence that task-orientated interventions such as Taekwondo may be useful [48, 49]. This sport is renowned for its swift kicks and fast movements which provide practice for single leg standing whilst maintaining body balance and enhancing postural control and sensory organisation in typically developing adolescents [48, 49]. Our review however found little evidence to support the use of traditional physical or occupational therapies, although these therapies were only assessed in one study included in our review. Interestingly, the recent European Academy for Childhood Disability DCD guidelines [11] which used a literature review and expert consensus approach to reach its conclusions, recommended only *task-orientated approaches* including a *cognitive component* [11] to improve motor skills in children with DCD. An example of this approach is the Cognitive Orientation to Daily Occupational Performance (CO OP) intervention recommended for children with DCD [11]. The child is trained to not only learn the motor task but monitor their own performance and self-evaluate the outcome [11].

Components of the task-orientated interventions included in this study which may explain their greater effect include the competitive nature of the task which ensures children naturally refine their gross motor skills to win. Also, their superior benefit may be explained by the use of cognitive skills for planning game strategy, and the fact that the tasks are fun, functional activities able to be integrated in daily life as leisure or recreation tasks. In addition, training was provided over a longer period of time (12 weeks), and a HEP with compliance measures was included. These strategies may ensure treatment dosage is optimised to enable motor learning and skill acquisition to occur. Wii Fit may not have shown the same success because the training period was very short (10 mins, 3 times per week over 4 weeks, PEDro score = 5) and Table Tennis because of poor methodology (PEDro score =3). These key components may be important when considering the effectiveness of intervention choices to address a child’s specific gross motor difficulties. Other considerations of intervention provision such as ideal frequency and duration, optimal intervention approach, age or gender considerations and the benefits of HEP’s were unable to be explored from the available evidence. Our review differs from others available as it only includes RCT’s, with interventions specifically aimed to improve gross motor performance, using meta-analysis to provide an overall treatment effect and GRADE rating to assess the believability of the meta-analysis findings.

Our group is frequently asked to manage mild to moderate gross motor disorders in children with FASD and this review was originally undertaken to determine suitable intervention strategies for them. However in the absence of clinical trials to guide effective treatment for children with FASD, the review extended to include RCT’s investigating similar mild to moderate gross motor disorders to inform efficacious treatment choice.

The surprising paucity of RCT’s (*n* = 9) means that the conclusions of this review have been drawn from a small study base. No study reported child satisfaction or cost effectiveness of interventions, both which may link to compliance. To evaluate the quality of included trial, sources of bias were identified by assessing methodological quality using the PEDro scale, which has been shown to have acceptable validity [30, 57] and reliability [31]. Bias may have been introduced from poor assessor blinding (4/9) and intention to treat analysis (3/9). In addition, the very small sample sizes of the trials included (range: 4 to 74) increases the risk of a type 2 error such that interventions fail to show an effect size when they have effectiveness. This review highlights challenges in conducting randomised intervention trials in paediatric populations. Authors of some excluded studies reported the initial RCT study design was changed in response to parents being unwilling to provide consent if their child received the control [58]. The small sample sizes also indicate the difficulties in locating children with similar diagnoses and the logistics of conducting intervention trials.

The strengths of this systematic review include the use of a registered prospective protocol and a highly sensitive search strategy to locate the best available evidence including hand searching of conference proceedings, clinical trial registries, relevant systematic reviews and approaching experts in the field for suitable references. Only trials of higher quality were included containing randomized interventions and a control group, data with a size effect and using international recognised criteria to diagnose participant inclusion. Given the lack of treatment effects, we explored the treatment effect for both the *most* and *least conservative* SMD. In addition, we assessed the overall quality of evidence using the GRADE approach, which has not been done in previous reviews.

Limitations of our review include use of only English-language trials and exclusion of unpublished clinical trials. Funnel plots were used to investigate small studies effects, which were found to be present in the *least conservative* estimate. This is likely due to one outlier with a small to moderate effect size (SMD:−2.7 (95% CI:−5.5 to 0.0) and the small participant numbers from which data in this CP trial was able to be extracted (*n* = 4) [47]. We acknowledge that only 9 studies rather than the recommended 10 studies were included [36] and the low power of these plots limits the conclusions that can be obtained [36]. We acknowledge that the quality of the data available to be extracted will limit the interpretation of the pooled effect and overall conclusions from our meta-analysis. In our review the limited trials available for meta-analyses meant we were not able to pool like intervention approaches to determine effectiveness of gross motor interventions. Also, many sources of heterogeneity existed including the variety of experimental interventions (*n* = 11), intervention approaches (*n* = 5), comparators (*n* = 3), age ranges (5–18 years) and standardized assessment tools (*n* = 6) however the meta-analyses are considered appropriate given that the highest *I* ^2^ value (0%) is less than the 25% threshold for low heterogeneity [37] reflecting homogeneity of the included trials effect sizes.

Task-orientated approaches are most effective for improving gross motor outcomes compared to other therapy approaches such as traditional, process-orientated or psychological. The evidence for task-orientated approaches would be further strengthened by replication in other intervention trials with strong methodical design. We recommend that future research should focus on RCT’s with larger sample sizes (*n* = 100), using motivating task-orientated therapy approaches including a cognitive component, with adequate training duration and frequency and containing a home exercise program to enhance motor learning. Gross motor outcomes should encompass measures such as (i) standardised assessment tools sensitive to change and able to measure components of skill acquisition targeted during intervention training and (ii) functional improvement in activity/participation. Short and long term follow-up points should be included to determine if skill acquisition is sustained. Other outcomes such as compliance, parental satisfaction, child satisfaction and cost should also be incorporated into the study design.

## Conclusion

The best available evidence from randomised trails suggests that some interventions improve gross motor performance in children with DCD or CP and that the effect is large. However the low quality of this evidence associated with methodological limitations of the trials, reduces our confidence to adopt these interventions and suggests the need for more rigorous trials. Interventions found to be most effective for motor learning and skill acquisition are interventions that have a task orientated approach and include reinforcement by a home exercise program and a compliance log. Given that mild to moderate gross motor disorders are common in many childhood conditions, high quality intervention trials are urgently needed to determine which interventions are most effective and which aspects of their delivery such as frequency and duration of therapy are important. This information will inform the management plans for children and guide allocation of limited physiotherapy and family resources.

## Additional files

Additional file 1:
**Data base literature searches.** (DOCX 34 kb)
Additional file 2:
**Inclusion Criteria Form.** (DOC 133 kb)
Additional file 3:
**Data Extraction Form.** (DOCX 81 kb)
Additional file 4:
**Funnel plot analysis of publication bias – most conservative SMD of trials included in meta-analysis.** (PDF 677 kb)
Additional file 5:
**Funnel plot analysis of publication bias – least conservative SMD of trials included in meta-analysis.** (PDF 685 kb)
Additional file 6:
**Calculation of size effects for comparison between cases and controls.** (DOCX 23 kb)
Additional file 7:
**List of excluded full-text articles and the primary reason for exclusion.** (DOCX 45 kb)

## Abbreviations

CI: Confidence Interval
CP: Cerebral Palsy
DCD: Developmental Coordination Disorder
FASD: Fetal Alcohol Spectrum Disorder
FES: Functional Electrical Stimulation
GM: Gross motor
GMFCS: Gross Motor Function Classification System
GRADE: Grading of Recommendations Assessment Development Evaluation
HEP: Home Exercise Program
PAE: Prenatal Alcohol Exposure
PEDro: Physiotherapy Evidence Database scale
RCT: Randomized Control Trial
SD: Standard Deviation
SE: Standard Error
SMD: Standard Mean Difference
