# Supplementary material for: Interventions to improve gross motor performance in children with neurodevelopmental disorders: a meta-analysis
Source: BMC Pediatr. 2016 Nov 29;16:193. doi: 10.1186/s12887-016-0731-6 (PMC5129231; doi:10.1186/s12887-016-0731-6)
Supplement: Additional file 1: — Data base literature searches. (DOCX 34 kb) [file 12887_2016_731_MOESM1_ESM.docx]

**Appendix 1 Data Base Literature Searches**

Completed 08.06.2015

**1. MEDLINE**

1. "Fetal Alcohol Spectrum Disorders"/

2. fasds.tw.

3. fetal alcohol spectrum disorder*.tw.

4. fetal alcohol syndrome*.tw.

5. Prenatal Exposure Delayed Effects/

6. partial fetal alcohol syndrome*.tw.

7. alcohol related neurodevelopmental disorder*.tw.

8. prenatal* alcohol expos*.tw.

9. fetal alcohol.tw.

10. Alcohol-Related Disorders/

11. Alcohol Drinking/

12. Pregnancy/

13. 11 and 12

14. or/1-13

15. infant, premature/

16. infant, extremely premature/

17. Premature Birth/

18. ((premature or preterm) adj birth*).tw.

19. preterm.tw.

20. (elbw or vlbw).tw.

21. infant, small for gestational age/

22. infant, very low birth weight/

23. infant, extremely low birth weight/

24. infant, low birth weight/

25. (infant* adj low birth weight).tw.

26. (infant* adj very low birth weight).tw.

27. (infant* adj extreme* low birth weight).tw.

28. or/15-27

29. Cerebral pals*.tw.

30. cerebral palsies.tw.

31. 29 or 30

32. Developmental coordination disorder*.tw

33. Clumsy child*.tw.

34. Minimal brain dysfunction.tw.

35. or/32-34

36. Brain injuries.tw.

37. Brain concussion.mp.

38. Brain haemorrhage, traumatic.mp.

39. Brain injury, chronic.mp.

40. Diffuse axonal injures.mp.

41. Hypoxia brain.mp.

42. ((Acquired or minimal) adj (brain injur*)).tw.

43. /36-42

44. developmental delay/

45. developmental delay.tw.

46. 44 or 45

47. Motor Skills/

48. motor skill*.tw.

49. gross motor skill*.tw.

50. motor Skills Disorders/

51. psychomotor Disorders/

52. psychomotor performance/

53. child development/

54. child development.tw.

55. cognitive motor development.tw.

56. dyspraxia.tw.

57. developmental dyspraxia.tw.

58. sensori-motor.tw.

59. sensorimotor.tw.

60. coordination.tw.

61. motor coordination.tw.

62. task specific training.tw.

63. task orientated training.tw.

64. balance games.tw.

65. balance.tw.

66. gait.tw.

67. agility.tw.

68. running speed*.tw.

69. bilateral coordination.tw.

70. muscle strength.tw.

71. ball skill*.tw.

72. (postural adj (stability or control)).tw.

73. or/47-72

74. exp physical therapy modalities/

75. physical therap*.tw.

76. physiotherap*.tw.

77. /74-76

78. child/

79. child, preschool/

80. (child* or preschool* or preschool* or schoolchild* or adolescent*).tw.

81. /78-80

82. 14 or 28 or 31 or 35 or 43 or 46

83. 73 and 77 and 81

84. 82 and 83

**2. PsycINFO**

1. "Fetal Alcohol Spectrum Disorders"/

2. fasds.tw.

3. fetal alcohol spectrum disorder*.tw.

4. fetal alcohol syndrome*.tw.

5. Prenatal Exposure Delayed Effects/

6. partial fetal alcohol syndrome*.tw.

7. alcohol related neurodevelopmental disorder*.tw.

8. prenatal* alcohol expos*.tw.

9. fetal alcohol.tw.

10. Alcohol-Related Disorders/

11. Alcohol Drinking/

12. Pregnancy/

13. 11 and 12

14. or/1-13

15. infant, premature/

16. infant, extremely premature/

17. Premature Birth/

18. ((premature or preterm) adj birth*).tw.

19. preterm.tw.

20. (elbw or vlbw).tw.

21. infant, small for gestational age/

22. infant, very low birth weight/

23. infant, extremely low birth weight/

24. infant, low birth weight/

25. (infant* adj low birth weight).tw.

26. (infant* adj very low birth weight).tw.

27. (infant* adj extreme* low birth weight).tw.

28. or/15-27

29. Cerebral pals*.tw.

30. cerebral palsies.tw.

31. 29 or 30

32. Developmental coordination disorder*.tw

33. Clumsy child*.tw.

34. Minimal brain dysfunction.tw.

35. or/32-34

36. Brain injuries.tw.

37. Brain concussion.mp.

38. Brain haemorrhage, traumatic.mp.

39. Brain injury, chronic.mp.

40. Diffuse axonal injures.mp.

41. Hypoxia brain.mp.

42. ((Acquired or minimal) adj (brain injur*)).tw.

43. /36-42

44. developmental delay/

45. developmental delay.tw.

46. 44 or 45

47. Motor Skills/

48. motor skill*.tw.

49. gross motor skill*.tw.

50. motor Skills Disorders/

51. psychomotor Disorders/

52. psychomotor performance/

53. child development/

54. child development.tw.

55. cognitive motor development.tw.

56. dyspraxia.tw.

57. developmental dyspraxia.tw.

58. sensori-motor.tw.

59. sensorimotor.tw.

60. coordination.tw.

61. motor coordination.tw.

62. task specific training.tw.

63. task orientated training.tw.

64. balance games.tw.

65. balance.tw.

66. gait.tw.

67. agility.tw.

68. running speed*.tw.

69. bilateral coordination.tw.

70. muscle strength.tw.

71. ball skill*.tw.

72. (postural adj (stability or control)).tw.

73. or/47-72

74. exp physical therapy modalities/

75. physical therap*.tw.

76. physiotherap*.tw.

77. /74-76

78. child/

79. child, preschool/

80. (child* or preschool* or preschool* or schoolchild* or adolescent*).tw.

81. /78-80

82. 14 or 28 or 31 or 35 or 43 or 46

83. 73 and 77 and 81

84. 82 and 83

**3. AMED**

1. "Fetal Alcohol Spectrum Disorders"/

2. fasds.tw.

3. fetal alcohol spectrum disorder*.tw.

4. fetal alcohol syndrome*.tw.

5. Prenatal Exposure Delayed Effects/

6. partial fetal alcohol syndrome*.tw.

7. alcohol related neurodevelopmental disorder*.tw.

8. prenatal* alcohol expos*.tw.

9. fetal alcohol.tw.

10. Alcohol-Related Disorders/

11. Alcohol Drinking/

12. Pregnancy/

13. 11 and 12

14. or/1-13

15. infant, premature/

16. infant, extremely premature/

17. Premature Birth/

18. ((premature or preterm) adj birth*).tw.

19. preterm.tw.

20. (elbw or vlbw).tw.

21. infant, small for gestational age/

22. infant, very low birth weight/

23. infant, extremely low birth weight/

24. infant, low birth weight/

25. (infant* adj low birth weight).tw.

26. (infant* adj very low birth weight).tw.

27. (infant* adj extreme* low birth weight).tw.

28. or/15-27

29. Cerebral pals*.tw.

30. cerebral palsies.tw.

31. 29 or 30

32. Developmental coordination disorder*.tw

33. Clumsy child*.tw.

34. Minimal brain dysfunction.tw.

35. or/32-34

36. Brain injuries.tw.

37. Brain concussion.mp.

38. Brain haemorrhage, traumatic.mp.

39. Brain injury, chronic.mp.

40. Diffuse axonal injures.mp.

41. Hypoxia brain.mp.

42. ((Acquired or minimal) adj (brain injur*)).tw.

43. /36-42

44. developmental delay/

45. developmental delay.tw.

46. 44 or 45

47. Motor Skills/

48. motor skill*.tw.

49. gross motor skill*.tw.

50. motor Skills Disorders/

51. psychomotor Disorders/

52. psychomotor performance/

53. child development/

54. child development.tw.

55. cognitive motor development.tw.

56. dyspraxia.tw.

57. developmental dyspraxia.tw.

58. sensori-motor.tw.

59. sensorimotor.tw.

60. coordination.tw.

61. motor coordination.tw.

62. task specific training.tw.

63. task orientated training.tw.

64. balance games.tw.

65. balance.tw.

66. gait.tw.

67. agility.tw.

68. running speed*.tw.

69. bilateral coordination.tw.

70. muscle strength.tw.

71. ball skill*.tw.

72. (postural adj (stability or control)).tw.

73. or/47-72

74. exp physical therapy modalities/

75. physical therap*.tw.

76. physiotherap*.tw.

77. /74-76

78. child/

79. child, preschool/

80. (child* or preschool* or preschool* or schoolchild* or adolescent*).tw.

81. /78-80

82. 14 or 28 or 31 or 35 or 43 or 46

83. 73 and 77 and 81

84. 82 and 83

**4. EMBASE**

1. "Fetal Alcohol Spectrum Disorders"/

2. fasds.tw.

3. fetal alcohol spectrum disorder*.tw.

4. fetal alcohol syndrome*.tw.

5. Prenatal Exposure Delayed Effects/

6. partial fetal alcohol syndrome*.tw.

7. alcohol related neurodevelopmental disorder*.tw.

8. prenatal* alcohol expos*.tw.

9. fetal alcohol.tw.

10. Alcohol-Related Disorders/

11. Alcohol Drinking/

12. Pregnancy/

13. 11 and 12

14. or/1-13

15. infant, premature/

16. infant, extremely premature/

17. Premature Birth/

18. ((premature or preterm) adj birth*).tw.

19. preterm.tw.

20. (elbw or vlbw).tw.

21. infant, small for gestational age/

22. infant, very low birth weight/

23. infant, extremely low birth weight/

24. infant, low birth weight/

25. (infant* adj low birth weight).tw.

26. (infant* adj very low birth weight).tw.

27. (infant* adj extreme* low birth weight).tw.

28. or/15-27

29. Cerebral pals*.tw.

30. cerebral palsies.tw.

31. 29 or 30

32. Developmental coordination disorder*.tw

33. Clumsy child*.tw.

34. Minimal brain dysfunction.tw.

35. or/32-34

36. Brain injuries.tw.

37. Brain concussion.mp.

38. Brain haemorrhage, traumatic.mp.

39. Brain injury, chronic.mp.

40. Diffuse axonal injures.mp.

41. Hypoxia brain.mp.

42. ((Acquired or minimal) adj (brain injur*)).tw.

43. /36-42

44. developmental delay/

45. developmental delay.tw.

46. 44 or 45

47. Motor Skills/

48. motor skill*.tw.

49. gross motor skill*.tw.

50. motor Skills Disorders/

51. psychomotor Disorders/

52. psychomotor performance/

53. child development/

54. child development.tw.

55. cognitive motor development.tw.

56. dyspraxia.tw.

57. developmental dyspraxia.tw.

58. sensori-motor.tw.

59. sensorimotor.tw.

60. coordination.tw.

61. motor coordination.tw.

62. task specific training.tw.

63. task orientated training.tw.

64. balance games.tw.

65. balance.tw.

66. gait.tw.

67. agility.tw.

68. running speed*.tw.

69. bilateral coordination.tw.

70. muscle strength.tw.

71. ball skill*.tw.

72. (postural adj (stability or control)).tw.

73. or/47-72

74. exp physical therapy modalities/

75. physical therap*.tw.

76. physiotherap*.tw.

77. /74-76

78. child/

79. child, preschool/

80. (child* or preschool* or preschool* or schoolchild* or adolescent*).tw.

81. /78-80

82. 14 or 28 or 31 or 35 or 43 or 46

83. 73 and 77 and 81

84. 82 and 83

**5. CINAHL**

1. "Fetal Alcohol Spectrum Disorders"/

2. fasds.tw.

3. fetal alcohol spectrum disorder*.tw.

4. fetal alcohol syndrome*.tw.

5. Prenatal Exposure Delayed Effects/

6. partial fetal alcohol syndrome*.tw.

7. alcohol related neurodevelopmental disorder*.tw.

8. prenatal* alcohol expos*.tw.

9. fetal alcohol.tw.

10. Alcohol-Related Disorders/

11. Alcohol Drinking/

12. Pregnancy/

13. 11 and 12

14. or/1-13

15. infant, premature/

16. infant, extremely premature/

17. Premature Birth/

18. ((premature or preterm) adj birth*).tw.

19. preterm.tw.

20. (elbw or vlbw).tw.

21. infant, small for gestational age/

22. infant, very low birth weight/

23. infant, extremely low birth weight/

24. infant, low birth weight/

25. (infant* adj low birth weight).tw.

26. (infant* adj very low birth weight).tw.

27. (infant* adj extreme* low birth weight).tw.

28. or/15-27

29. Cerebral pals*.tw.

30. cerebral palsies.tw.

31. 29 or 30

32. Developmental coordination disorder*.tw

33. Clumsy child*.tw.

34. Minimal brain dysfunction.tw.

35. or/32-34

36. Brain injuries.tw.

37. Brain concussion.mp.

38. Brain haemorrhage, traumatic.mp.

39. Brain injury, chronic.mp.

40. Diffuse axonal injures.mp.

41. Hypoxia brain.mp.

42. ((Acquired or minimal) adj (brain injur*)).tw.

43. /36-42

44. developmental delay/

45. developmental delay.tw.

46. 44 or 45

47. Motor Skills/

48. motor skill*.tw.

49. gross motor skill*.tw.

50. motor Skills Disorders/

51. psychomotor Disorders/

52. psychomotor performance/

53. child development/

54. child development.tw.

55. cognitive motor development.tw.

56. dyspraxia.tw.

57. developmental dyspraxia.tw.

58. sensori-motor.tw.

59. sensorimotor.tw.

60. coordination.tw.

61. motor coordination.tw.

62. task specific training.tw.

63. task orientated training.tw.

64. balance games.tw.

65. balance.tw.

66. gait.tw.

67. agility.tw.

68. running speed*.tw.

69. bilateral coordination.tw.

70. muscle strength.tw.

71. ball skill*.tw.

72. (postural adj (stability or control)).tw.

73. or/47-72

74. exp physical therapy modalities/

75. physical therap*.tw.

76. physiotherap*.tw.

77. /74-76

78. child/

79. child, preschool/

80. (child* or preschool* or preschool* or schoolchild* or adolescent*).tw.

81. /78-80

82. 14 or 28 or 31 or 35 or 43 or 46

83. 73 and 77 and 81

84. 82 and 83

**6. PEDro**

1. pediatrics and motor

2. brain injuries

3. brain haemorrhage

4. brain concussion

5. hypoxic brain

6. brain injury and motor

7. fetal alcohol spectrum disorder

8. fetal alcohol syndrome

9. developmental co-ordinaation disorder

10. dyspraxia

11. sensorimotor and children

12. balance and children

13. premature infants and motor

14. preterm infants and motor

15. extremely low birth weight and motor

16. developmental delay and motor

17. cerebral palsy and motor skills

18. physiotherapy and motor skills

19. physical therapy and motor skills

20. infants and motor skills

21. child and motor skills

22. adolescent and motor skills

23. gross motor skills

24. motor skills

24. interventions and motor skills

**7. Google Scholar**

1. brain injury and motor skills/gross motor skills/ physiotherapy/physical therapy

2. brain haemorrhage and motor skills/ gross motor skills/ physiotherapy/physical therapy

3. brain concussion and motor skills/ gross motor skills/ physiotherapy/physical therapy

4. hypoxia brain injury and motor skills/ gross motor skills/ physiotherapy/physical therapy

5. fetal alcohol spectrum disorder and motor skills/gross motor skills/physiotherapy/physical therapy

6. fetal alcohol syndrome and motor skills/gross motor skills/physiotherapy/physical therapy

7. developmental co-ordination disorder and motor skills/gross motor skills/physiotherapy/physical therapy

8. dyspraxia and motor skills/gross motor skills/physiotherapy/physical therapy

9. sensorimotor and motor skills/gross motor skills/physiotherapy/physical therapy

10. balance and motor skills/gross motor skills/physiotherapy/physical therapy

11. premature infants and motor skills/gross motor skills/physiotherapy/physical therapy

12. preterm infants and motor skills/gross motor skills/physiotherapy/physical therapy

13. extremely low birth weight and motor skills/gross motor skills/physiotherapy/physical therapy

14. developmental delay and motor skills/gross motor skills/physiotherapy/physical therapy

15. cerebral palsy and motor skills/gross motor skills/physiotherapy/physical therapy

16. physiotherapy and motor skills/gross motor skills

17. physical therapy and motor skills/gross motor skills

18. infants and motor skills/gross motor skills/physiotherapy/physical therapy

19. child and motor skills/gross motor skills/physiotherapy/physical therapy

20. adolescent and motor skills/gross motor skills/physiotherapy/physical therapy

21. interventions and motor skills/gross motor skills/physiotherapy/physical therapy

**8. The Cochrane Collaboration**

1. brain injuries

2. brain haemorrhage

3. brain concussion

4. hypoxia brain

5. brain injury and motor

6. fetal alcohol spectrum disorder

7. fetal alcohol syndrome

8. developmental co-ordination disorder

9. dyspraxia

10. sensorimotor

11. balance

12. premature infants and motor

13. preterm infants and motor

14. extremely low birth weight and motor

15. developmental delay and motor

16. cerebral palsy and motor

17. physiotherapy and motor

18. physical therapy and motor

19. infants and motor

20. child and motor

21. adolescent and motor

22. gross motor

23. motor skills

24. interventions and motor

**9. Clinical Trial Registry Searches**

Both ANZCTR and WHO CTRP searched

1. fetal alcohol spectrum disorders

2. fetal alcohol syndrome

3. developmental co-ordination disorder

4. cerebral palsy

5. preterm infant

6. premature infant

7. brain injury

8. brain trauma

9. developmental delay

10. gross motor delay

11. gross motor

12. motor skills

13. physiotherapy

14. physical therapy
