# Supplementary material for: Interventions to improve gross motor performance in children with neurodevelopmental disorders: a meta-analysis
Source: BMC Pediatr. 2016 Nov 29;16:193. doi: 10.1186/s12887-016-0731-6 (PMC5129231; doi:10.1186/s12887-016-0731-6)
Supplement: Additional file 2: — Inclusion Criteria Form. (DOC 133 kb) [file 12887_2016_731_MOESM2_ESM.doc]

| First author: | | Year: |
| --- | --- | --- |
| 1. STUDY DESIGN | |  |
| a) Study design is not a case study, review, reliability OR validity study | | Yes  No  Uncertain |
| b) STUDY DESIGN: Human based intervention which contains a control group:   - RCT and non-randomised clinical trial - Quasi RCT (contains control group)   **Control includes no treatment, waiting list, usual therapy and placebo* | | Yes  No  Uncertain  Comments: |
| 2. PARTICIPANTS | |  |
| a) Participants were 3-18 years as reported in the study inclusion criteria?  (ie: range of children aged from 3 to 18yrs) | | Yes  No  Uncertain  Comments: |
| b) Diagnosis: Must answer yes to 1 only: i, ii, iii, iv or v  Must answer yes to vi | |  |
|  | i) Fetal Alcohol Spectrum Disorders determined using internationally recognised standardised diagnostic criteria (listed below):   - Fetal alcohol Syndrome (FAS) - partial - Fetal Alcohol Syndrome (p-FAS) - Alcohol Related Neurodevelopmental Disorders (ARND)   ** Recognized criteria: UniW, IOM, CDC, Canadian Guidelines or other)* | Yes  No  Uncertain  Comments: |
|  | ii) Developmental Coordination Disorder determined using internationally recognised standardised diagnostic criteria as defined by the DSM IV or V | Yes  No  Uncertain  Comments: |
|  | iii) Mild – moderate gross motor disorders in preterm infant population (≤ 28 weeks gestational age, < 1000g) and discharged form hospital | Yes  No  Uncertain  Comments: |
|  | iv) Acquired minimal brain injury or mild traumatic brain injury defined by GCS ≥ 13 | Yes  No  Uncertain  Comments: |
|  | v) Cerebral Palsy as defined by GMFCS 1- dipegia, hemiplegia and quadriplegia  **Level 1 defined as “children walk indoors and outdoors, and climb stairs without limitations. Children perform gross motor skills including running and jumping but speed, balance, and coordination are reduced”.*  CP interventions do not consist of the following:   - *Treadmill training to improve gait without a balance outcome Constraint induced therapy – for hemiplegia (unilateralspasticity)* - *Botox therapy – pharmacological therapy to reduce spasticity* - *Robot treatment – appropriate forseverely impaired CP children* - *Suit wearing – to splint children with poor postural control due to spasticity* | Yes  No  Uncertain  Comments: |
|  | V) Subjects do not have any of the following conditions:   - Spasticity and dystonia - Hip dysplasia (ie developmental dysplasia of hips – DDH) - Chromosomal disorders/Syndromes – eg Downs, Angleman etc | Yes  No  Uncertain  Comments: |
| 3. INTERVENTION | |  |
| a)_Intervention being investigated is NOT one of the following:  *• Surgical interventions*  *• Electrical/EMG interventions*   - *Pharmacological* | | Yes  No  Uncertain  Comments: |
| 4. ASSESMENT AND OUTCOMES | |  |
| a) Gross motor outcome assessed using a standardized measure and reported?  **Gross motor outcome: measures of the function of large muscle groups. Domains assessed might include strength, endurance, balance, co-ordination, agility, ball skills, power, head control, core stability.*  **Exclude outcomes self-reported by mother / caregiver(as this may be a source of bias)*  **Exclude measurements using the “manual ability classification system (MACS)– generally thought of as a fine motor measure, similar to the GMFCS but for upper limbs* | | Yes  No  Uncertain  Comments: |
| b) Statistical comparisons conducted for intervention and control group:   - Quantitative size effect plus - Standard error (SE) OR - Standard deviation (SD) OR - Confidence interval (CI)   **Study needs to provide quantitative measures of gross motor function. Simply reporting a p value is insufficient.* | | Yes  No  Uncertain  Comments: |
| **Study meets inclusion criteria?**  All questions above should be ‘yes’ for inclusion   - 2b should include 1 yes from i-v | | Yes  No  Uncertain  Comments: |
| Reviewer: | | Barb Lucas  Sarah Coggan |


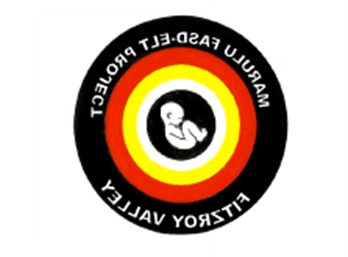
**Inclusion criteria form**
