# Supplementary material for: Interventions to improve gross motor performance in children with neurodevelopmental disorders: a meta-analysis
Source: BMC Pediatr. 2016 Nov 29;16:193. doi: 10.1186/s12887-016-0731-6 (PMC5129231; doi:10.1186/s12887-016-0731-6)
Supplement: Additional file 3: — Data Extraction Form. (DOCX 81 kb) [file 12887_2016_731_MOESM3_ESM.docx]

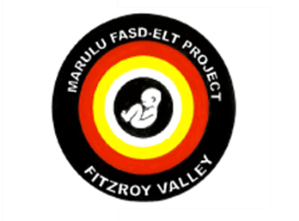


Rater: ________________________ Article No: ________________________

| **CATEGORY** | **COMMENT** | |
| --- | --- | --- |
| **Authors :**  **Study Year: Study Location (Country):** | | |
| **Study Type**  **(please circle)** | RCT Quasi RCT Cohort Other: please identify______________ | |
| **Mean Age (total)** |  | |
| **Sample Size (total)** |  | |
| **Participant** **Characteristics** | Diagnosis (please tick applicable box) | Fetal Alcohol Spectrum Disorders  Developmental Coordination Disorder  Mild – moderate GM disorders in preterm infants  Minimal brain injury / mild traumatic brain injury  Cerebral Palsy (Level 1)  Developmental Delay  GM Impairment ≥ -1SD (16^th^ centile) |
|  | Diagnostic criteria utilised |  |
|  | Number of participants |  |
|  | Gender breakdown of participants |  |
|  | Age breakdown of participants | 0-2years  5- <10 yrs  2- <5 yrs  10 - < 18 yrs |
|  | Other comments |  |
| **Reference Group**  **Characteristics** | General description of selection method |  |
|  | Number of participants |  |
|  | Gender breakdown of participants |  |
|  | Age breakdown of participants | 0-2years  5- <10 yrs  2- <5 yrs  10 - < 18 yrs |
| **Intervention: Group and treatment description** | Treatment approach and setting |  |
|  | Treatment duration, frequency and intensity |  |
|  | Protocol compliance |  |
|  | Standardised Assessment Tool for Gross Motor outcome |  |
|  | Measurement of change: |  |
| **CATEGORY** | **COMMENT** | |
| **Comparison: Group and Treatment description** | Treatment approach and setting |  |
|  | Treatment duration, frequency and intensity |  |
|  | Protocol compliance |  |
|  | Standardised Assessment Tool for Gross Motor outcome |  |
|  | Measurement of change: |  |
| **Assessor’s Profession**  (eg PT, Psych, doctor etc) |  | |
| **Gross Motor Outcomes Measure:** | Areas significantly affected compared with control |  |
|  | Prevalence of Gross Motor impairment |  |
|  | Mean scores and SDs /SEs |  |
|  | GM outcome: Direction of effect |  |
|  | Statistical Analysis methods used |  |
|  | Estimate effect of statistical analysis related to GM impairment |  |
|  | Confounders controlled for in Gross Motor statistical analysis |  |
| **Secondary Outcomes:** | Compliance |  |
|  | Parental satisfaction |  |
|  | Patient satisfaction |  |
|  | Cost effectiveness |  |
