# Supplementary figures and images for: Interventions to improve gross motor performance in children with neurodevelopmental disorders: a meta-analysis
Source: BMC Pediatr. 2016 Nov 29;16:193. doi: 10.1186/s12887-016-0731-6 (PMC5129231; doi:10.1186/s12887-016-0731-6)

Most Conservative estimates

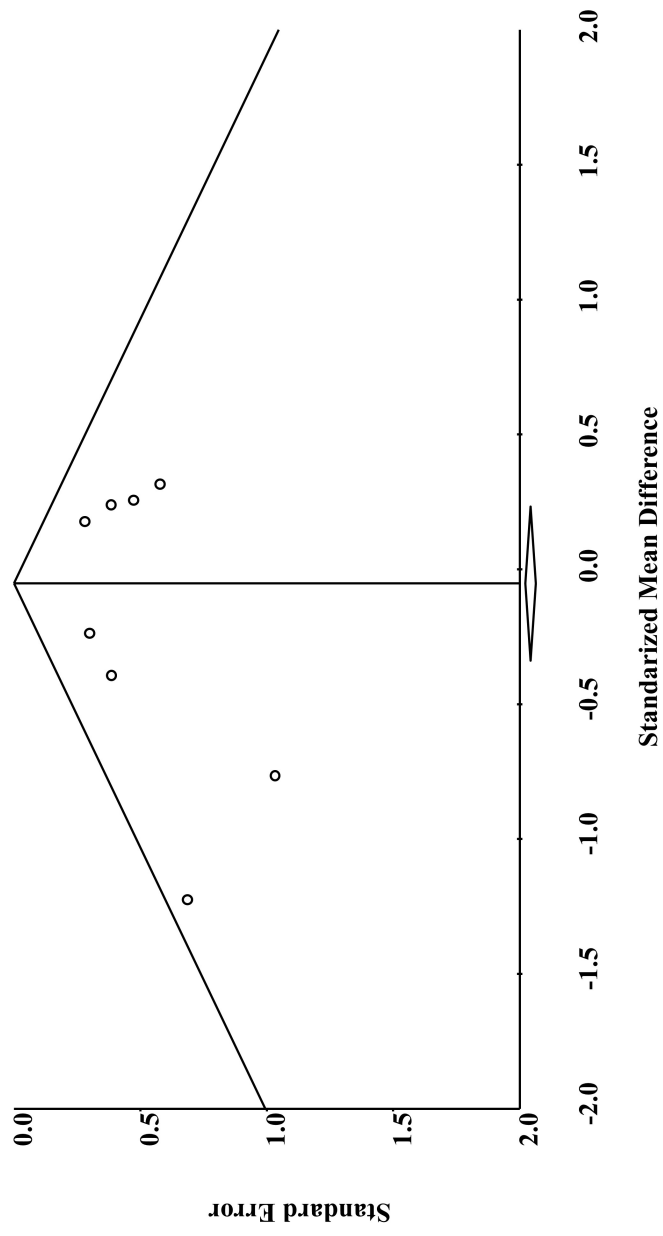

Supplement: Additional file 4: — Funnel plot analysis of publication bias – most conservative SMD of trials included in meta-analysis. (PDF 677 kb) [file 12887_2016_731_MOESM4_ESM.pdf]

Least Conservative estimates

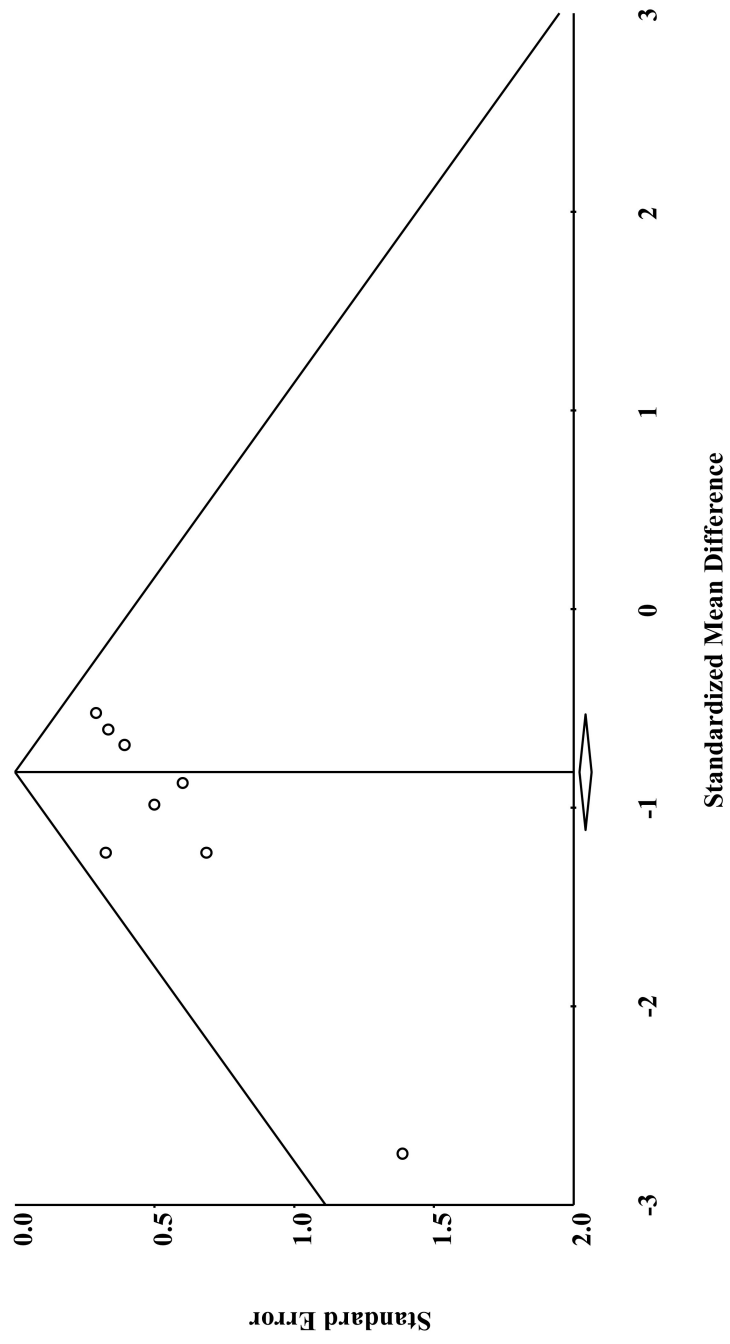

Supplement: Additional file 5: — Funnel plot analysis of publication bias – least conservative SMD of trials included in meta-analysis. (PDF 685 kb) [file 12887_2016_731_MOESM5_ESM.pdf]
