# Supplementary material for: Interventions to improve gross motor performance in children with neurodevelopmental disorders: a meta-analysis
Source: BMC Pediatr. 2016 Nov 29;16:193. doi: 10.1186/s12887-016-0731-6 (PMC5129231; doi:10.1186/s12887-016-0731-6)
Supplement: Additional file 6: — Calculation of size effects for comparison between cases and controls. (DOCX 23 kb) [file 12887_2016_731_MOESM6_ESM.docx]

**Appendix 4.** Calculation of size effects for comparison between cases and controls

| **Participant**  **type** | **Study reference** | **Intervention** | **Comparison** | **GM Outcome** | **Mean** | **SD** | **Intervention sample size** | **Mean** | **SD** | **Control sample size** | **SMD** |
| --- | --- | --- | --- | --- | --- | --- | --- | --- | --- | --- | --- |
| **1. CP** | aaChrysagis et al 2012 | Treadmill training | Usual care | *GMFM | 90.84 | 6.23 | 2 | 86.59 | 4.84 | 2 | -0.8 (-2.8 to 1.3) |
|  | abChrysagis et al 2012 | Treadmill training | Usual care | *Walking speed | 63.34 | 4.72 | 2 | 52.28 | 3.22 | 2 | 2.7 (-5.5 to 0.0) |
|  | bLedebt 2005 | Balance training | No treatment | *Balance: % of time Centre of Pressure on target in quiet standing | 83.75 | 11.25 | 5 | 70 | 11.25 | 5 | -1.2 (-2.6 to 0.1) |
| **2. DCD** | cFong et al 2012 | Taekwondo | No treatment | Unilateral Stance Test: Centre of Pressure sway velocity (non-dominant leg) | 2.21 | 1.88 | 21 | 3.79 | 1.77 | 23 | -0.9 (-1.5 to -0.2) |
|  | daFong et al 2013 | Taekwondo | No treatment | Motor Control Test: reactive balance control | 128.54 | 44.76 | 21 | 137.67 | 33.02 | 23 | -0.2 (-.0.8 to 0.4) |
|  | dbFong et al 2013 | Taekwondo | No treatment | Unilateral Stance Test: Centre of Pressure sway velocity (dominant leg) | 1.46 | 0.46 | 21 | 3.6 | 2.38 | 23 | -1.2 (-1.9 to -0.6) |
|  | eaHillier et al 2010 | Aquatic therapy | Waiting list | M-ABC: Ball skills | 3.83 | 2.86 | 6 | 3.08 | 1.69 | 6 | 0.3 (-0.8 to 1.5) |
|  | ebHillier et al 2010 | Aquatic therapy | Waiting list | M-ABC: Static/Dynamic balance | 4.92 | 2.89 | 6 | 8.25 | 4.57 | 6 | -0.9 (-2.1 to 0.3) |
|  | gaPeens et al 2008 | Motor intervention | No treatment | M-ABC: Ball skills  (Group 1) | 1.95 | 2.74 | 20 | 1.82 | 2.93 | 17 | 0.0 (-0.6 to 0.7) |
|  | gbPeens et al 2008 | Psych intervention | No treatment | M-ABC: Ball skills  (Group 2) | 1.7 | 1.21 | 10 | 1.82 | 2.93 | 17 | -0.0 (-0.8 to 0.7) |
|  | gcPeens et al 2008 | Psychomotor intervention | No treatment | M-ABC: Ball skills  (Group 3) | 2.55 | 3.12 | 11 | 1.82 | 2.93 | 17 | 0.2 (-0.5 to 1.0) |
|  | gdPeens et al 2008 | Motor intervention | No treatment | M-ABC: Static/Dynamic balance  (Group 1) | 1.18 | 1.08 | 20 | 2.5 | 3.02 | 17 | -0.6 (-1.3 to 0.1) |
|  | gePeens et al 2008 | Psych intervention | No treatment | M-ABC: Static/Dynamic balance  (Group 2) | 2.35 | 2.66 | 10 | 2.5 | 3.02 | 17 | -0.1 (-0.8 to 0.7) |
|  | gfPeens et al 2008 | Psychomotor intervention | No treatment | M-ABC: Static/Dynamic balance  (Group 3) | 1.05 | 3.47 | 11 | 2.5 | 3.02 | 17 | -0.5 (-1.2 to 0.3) |
|  | haPolatajko 1995 | Kinaesthetic Training | No treatment | TOMI: ball skills (Group 1) | 2.17 | 1.36 | 26 | 2.15 | 1.35 | 24 | 0.0 (-0.5 to 0.6) |
|  | hbPolatajko 1995 | Traditional intervention | No treatment | TOMI: ball skills (Group 2) | 1.48 | 1.23 | 24 | 2.15 | 1.35 | 24 | -0.5 (-1.1 to 0.1) |
| **Participant**  **type** | **Study reference** | **Intervention** | **Comparison** | **GM Outcome** | **Mean** | **SD** | **Intervention sample size** | **Mean** | **SD** | **Control sample size** | **SMD** |
|  | hcPolatajko 1995 | Kinaesthetic Training | No treatment | TOMI: static/dynamic balance (Group 1) | 3.19 | 1.64 | 26 | 2.89 | 1.69 | 24 | 0.2 (-0.4 to 0.7) |
|  | hdPolatajko 1995 | Traditional intervention | No treatment | TOMI: static/dynamic balance (Group 2) | 3.02 | 1.33 | 24 | 2.89 | 1.69 | 24 | 0.1 (-0.5 to 0.7) |
|  | iaTsai 2009 | Table tennis | No treatment | M-ABC: Ball skills | 4.69 | 3.09 | 13 | 6.5 | 2.2 | 14 | -0.7 (-1.5 to 0.1) |
|  | ibTsai 2009 | Table tennis | No treatment | M-ABC: Static/Dynamic balance | 3.5 | 1.85 | 13 | 4.25 | 1.99 | 14 | -0.4 (-1.2 to 0.4) |
|  |  |  |  |  | Group A | | | Group B | | |  |
|  | jaHammond et al 2013 | Wii Fit (Group A) | Usual care | *BOT2: Bilateral Coordination | 6.4 | 0.91 | 10 | 5.5 | 0.93 | 8 | 1.0 (-0.0 to 2.0) |
|  | jbHammond et al 2013 | Wii Fit (Group A) | Usual care | *BOT2: Balance | 7.8 | 2.91 | 10 | 6.88 | 1.46 | 8 | -0.4 (-1.4 to 0.5) |
|  | jcHammond et al 2013 | Wii Fit (Group A) | Usual care | *BOT2: Running speed & agility | 2.3 | 3.86 | 10 | 1.5 | 0.53 | 8 | -0.3 (-1.2 to 0.6) |
|  | jdHammond et al 2013 | Wii Fit (Group A) | Usual care | *BOT2: Upper limb coordination | 8.9 | 2.43 | 10 | 9.13 | 1.89 | 8 | 0.1 (-0.8 to 1.0) |
|  | jeHammond et al 2013 | Wii Fit (Group A) | Usual care | *BOT2: Strength | 6.8 | 1.93 | 10 | 7.63 | 4.31 | 8 | 0.2 (-0.7 to 1.2) |

* Higher scores indicate better performance

CP = cerebral palsy

DCD = developmental co-ordination disorder
