# Supplementary material for: Interventions to improve gross motor performance in children with neurodevelopmental disorders: a meta-analysis
Source: BMC Pediatr. 2016 Nov 29;16:193. doi: 10.1186/s12887-016-0731-6 (PMC5129231; doi:10.1186/s12887-016-0731-6)
Supplement: Additional file 7: — List of excluded full-text articles and the primary reason for exclusion. (DOCX 45 kb) [file 12887_2016_731_MOESM7_ESM.docx]

**Appendix 7: List of excluded full-text articles and the primary reason for exclusion**

| **#** | **Study** | **Title** |
| --- | --- | --- |
| **Participants not classified using GMFCS (n=32)** | | |
| 1 | Akhtina T et al 2003 | Improving spatial functioning in children with CP using computerised and traditional games tasks. |
| 2 | Al-Oraibi S et al 2006 | Implementation of constraint-induced movement therapy for young children with unilateral cerebral palsy in Jordan: a home-based model. |
| 3 | Bower E et al 1996 | A randomised controlled trial of different intensities of physiotherapy and different goal-setting procedures in 44 children with cerebral palsy. |
| 4 | Bumin G et al 2001 | Effectiveness of two different sensory-integration programmes for children with spastic diplegic cerebral palsy. |
| 5 | Carlsen P 1975 | Comparison of two occupational therapy approaches for treating the young cerebral palsied child. |
| 6 | Case-Smith J | Multicenter randomized controlled trial of pediatric constraint-induced movement therapy: 6-month follow-up. |
| 7 | Chan NN et al 2004 | Efficacy of neuromuscular electrical stimulation on improving ankle kinetics in walking on children with cerebral palsy. |
| 8 | Cherng R et al 2004 | The effectiveness of therapeutic horseback riding in children with spastic cerebral palsy. |
| 9 | Choi M et al 2011 | Effect of task-oriented training and neurodevelopmental treatment on the sitting posture in children with cerebral palsy |
| 10 | Choudhary A et al 2013 | Efficacy of modified constraint induced movement therapy in improving upper limb function in children with hemiplegic cerebral palsy: A randomized controlled trial. |
| 11 | Dali C 2002 | Threshold electrical stimulation (TES) in ambulant children with CP: a randomized, double blind placebo controlled trial. |
| 12 | Dresen MH et al 1985 | Aerobic energy expenditure of handicapped children after training. |
| 13 | Emara HA et al 2015 | Effect of a new physical therapy concept on dynamic balance in children with spastic cerebral palsy |
| 14 | Facchin P et al 2011 | Multisite trial comparing the efficacy of constraint-induced movement therapy with that of bimanual intensive training in children with hemiplegic cerebral palsy: post-intervention results. |
| 15 | Fedrizzi E et al 2013 | Unimanual and bimanual intensive training in children with hemiplegic cerebral palsy and persistence in time of hand function improvement: 6-month follow-up results of a multisite clinical trial. |
| 16 | Hamed NS et al 2011 | Pedometer-based gait training in children with spastic hemiparetic cerebral palsy: a randomized controlled study. |
| 17 | Hernandez-Reif M 2005 | Cerebral palsy symptoms in children decreased following massage therapy. |
| 18 | Hsin YJ et al 2012 | Efficacy of constraint-induced therapy on functional performance and health-related quality of life for children with cerebral palsy: a randomized controlled trial. |
| 19 | Ibrahim MM et al 2014 | Effect of whole-body vibration on muscle strength, spasticity, and motor performance in spastic diplegic cerebral palsy children |
| 20 | Kang H et al 2012 | Effects of hippotherapy on the sitting balance of children with cerebral palsy: A randomized control trial. |
| 21 | Lee BK et al 2013 | Effect of whole body vibration training on mobility in children with cerebral palsy: a randomized controlled experimenter-blinded study. |
| 22 | Lee CW et al 2014 | The effects of hippotherapy and a horse riding simulator on the balance of children with cerebral palsy. |
| 23 | Ketelaar et al 2001 | Effects of a functional therapy program on motor abilities of children with cerebral palsy |
| 24 | MacKinnon JR 1995 | A study of therapeutic effects of horseback riding for children with cerebral palsy |
| 25 | McGee MC et al 2009 | Immediate effects of a hippotherapy session on gait parameters in children with spastic cerebral palsy. |
| 26 | Pandey DP et al 2011 | Effect of functional strength training on functional motor performance in young children with cerebral palsy. |
| 27 | Park ES 2001 | The effect of electrical stimulation on the trunk control in young children with spastic cerebral palsy. |
| 28 | Reid S et al 2010 | Neuromuscular adaptations to eccentric strength training in children and adolescents with cerebral palsy. |
| 29 | Stiller C et al 2003 | The effect of conductive education, intensive therapy, and special education services on motor skills in children with cerebral palsy |
| 30 | Taub E et al 2004 | Efficacy of constraint-induced movement therapy for children with cerebral palsy with asymmetric motor impairment. |
| 31 | van den Berg-Emons RJ 1998 | Physical training of school children with spastic cerebral palsy: effects on daily activity, fat mass and fitness. |
| 32 | van der Linden ML 2003 | Electrical stimulation of gluteus maximus in children with cerebral palsy: effects on gait characteristics and muscle strength. |
| **Results not stratified to GMFCS 1 (n=41)** | | |
| 1 | Bohm H et al 2015 | Effects of climbing therapy on gait function in children and adolescents with cerebral palsy – a randomised, controlled cross-over |
| 2 | Borges MBS et al 2011 | Therapeutic effects of a horse riding simulator in children with cerebral palsy. |
| 3 | Chen CL et al 2012 | Muscle strength enhancement following home-based virtual cycling training in ambulatory children with cerebral palsy. |
| 4 | Cherng R-J et al 2007 | Effect of treadmill training with body weight support on gait and gross motor function in children with spastic cerebral palsy. |
| 5 | Christiansen AS et al 2008 | Intermittent versus continuous physiotherapy in children with cerebral palsy. |
| 6 | Crompton J et al 2007 | Group-based task-related training for children with cerebral palsy: a pilot study. |
| 7 | Davis E et al 2009 | A randomized controlled trial of the impact of therapeutic horse riding on the quality of life, health, and function of children with cerebral palsy. |
| 8 | de Brito Brandao M et al 2010 | Adapted version of constraint-induced movement therapy promotes functioning in children with cerebral palsy: a randomized controlled trial. |
| 9 | Dimitrijevic L et al 2012 | The effect of aquatic intervention on the gross motor function and aquatic skills in children with cerebral palsy. |
| 10 | Druzbicki M et al 2013 | Functional effects of robotic-assisted locomotor treadmill therapy in children with cerebral palsy. |
| 11 | Duncan B et al 2012 | Evaluating intense rehabilitative therapies with and without acupuncture for children with cerebral palsy: a randomized controlled trial. |
| 12 | el-Basatiny HMY et al 2014 | Effect of backward walking training on postural balance in children with hemiparetic cerebral palsy: a randomized controlled |
| 13 | el-Basatiny HMY et al 2014 | Effect of postural balance training on gait parameters in children with cerebral palsy. |
| 14 | el-Shamy SM et al 2014 | Effect of balance training on postural balance control and risk of fall in children with diplegic cerebral palsy [with consumer summary]. |
| 15 | El-Shamy S 2014 | El-Shamy SM. Effect of whole-body vibration on muscle strength and balance in diplegic cerebral palsy: a randomized controlled trial. |
| 16 | Engsberg JR et al 2006 | Increasing ankle strength to improve gait and function in children with cerebral palsy: a pilot study. |
| 17 | Fowler EG et al 2010 | Pediatric endurance and limb strengthening (PEDALS) for children with cerebral palsy using stationary cycling: a randomized controlled trial. |
| 18 | Grecco LA et al 2013 | A comparison of treadmill training and overground walking in ambulant children with cerebral palsy: randomized controlled clinical trial. |
| 19 | Grecco LA et al 2013 | Effect of treadmill gait training on static and functional balance in children with cerebral palsy: a randomized controlled trial. |
| 20 | Herrero P et al 2012 | Herrero P, Gomez-Trullen EM, Asensio A, Garcia E, Casas R, Monserrat E, et al. Study of the therapeutic effects of a hippotherapy simulator in children with cerebral palsy: a stratified single-blind randomized controlled trial. |
| 21 | Kang BS et al 2007 | Effects of botulinum toxin A therapy with electrical stimulation on spastic calf muscles in children withcerebral palsy. |
| 22 | Kara OK et al 2015 | The effects pf Kinesio Taping on body functions and activity in unilateral spastic cerebral palsy: a single blind randomised controlled trial |
| 23 | Katz-Leurer M et al 2009 | The effects of a 'home-based' task-oriented exercise programme on motor and balance performance in children with spastic cerebral palsy and severe traumatic brain injury. |
| 24 | Kerr C et al 2006 | Electrical stimulation in cerebral palsy:a randomized controlled trial. |
| 25 | Liao H et al 2007 | Effectiveness of loaded sit-to-stand resistance exercise for children with mild spastic diplegia: a randomized clinical trial. |
| 26 | Law MC et al 2011 | Focus on function: A cluster, randomized controlled trial comparing child-versus context-focused intervention for young children with cerebral palsy. |
| 27 | Mattern-Baxter K et al 2013 | Effects of home-based locomotor treadmill training on gross motor function in young children with cerebral palsy: a quasi-randomized controlled trial. |
| 28 | Morton J et al 2005 | The effects of progressive resistance training for children with cerebral palsy. |
| 29 | Olama KA et al 2012 | Effect of vibration versus suspension therapy on balance in children with hemiparetic cerebral palsy. |
| 30 | Park ES et al 2001 | The effect of electrical stimulation on the trunk control in young children with spastic cerebral palsy. |
| 31 | Park ES et al 2014 | Effects of hippotherapy on gross motor function and functional performance of children with cerebral palsy. |
| 32 | Patikas, D et al 2006a | Effects of a post-operative resistive exercise program on the knee extension and flexion torque in childern with cerebral palsy |
| 33 | Salem et al 2012 | Effectiveness of a low-cost virtual reality system for children with developmental delay: a preliminary randomised single-blind controlled trial |
| 34 | Scholtes VA et al 2012 | Effectiveness of functional progressive resistance exercise training on walking ability in children with cerebral palsy: a randomized controlled trial. |
| 35 | Shurtleff TL et al 2009 | Changes in dynamic trunk/ head stability and functional reach after hippotherapy. |
| 36 | Shurtleff TL et al 2010 | Changes in trunk and head stability in children with cerebral palsy after hippotherapy: a pilot study |
| 37 | Sterba JA et al 2007 | Horseback riding in children with cerebral palsy: effect on gross motor function. |
| 38 | Tsorlakis N 2004 | Effect of intensive neurodevelopmental treatment in gross motor function of children with cerebral palsy. |
| 39 | Wallen M et al 2011 | Modified constraint-induced therapy for children with hemiplegic cerebral palsy: a randomized trial. |
| 40 | Verschuren O et al 2007 | Exercise training program in children and adolescents with cerebral palsy: a randomized controlled trial. |
| 41 | Wang TH et al 2013 | A home-based program using patterned sensory enhancement improves resistance exercise effects for children with cerebral palsy: a randomized controlled trial. |
| **Participants don’t meet inclusion criteria (n=12)** | | |
| 1 | Bailes AF et al 2011 | The effect of suit wears during an intensive therapy program in children with cerebral palsy. |
| 2 | Bar-Haim S et al 2008 | Bar-Haim S, Harries N, Belokopytov M, Lahat E, Kaplanski J. Random perturbation: A potential aid in treatment of children with cerebral palsy. Disability and rehabilitation 2008; 30(19):1420-28. |
| 3 | Braga LW et al 2005 | Direct clinician-delivered versus indirect family-supported rehabilitation of children with traumatic brain injury: a randomized controlled trial. |
| 4 | Brunner AL et al 2014 | Continuous vs. blocks of physiotherapy for motor development in children with cerebral palsy and similar syndromes: A prospective randomized study. |
| 5 | Cicerone KD et al 2008 | A randomized controlled trial of holistic neuropsychologic rehabilitation after traumatic brain injury. |
| 6 | Dodd K et al 2003 | A randomized clinical trial of strength training in young people with cerebral palsy. |
| 7 | Druzbicki M et al 2010 | Assessment of the impact of orthotic gait training on balance in children with cerebral palsy. |
| 8 | Druzbicki M et al 2013 | Functional effects of robotic-assisted locomotor treadmill therapy in children with cerebral palsy. |
| 9 | Kaplan BJ 1993 | Reexamination of sensory integration treatment: A combination of 2 efficacy studies. |
| 10 | Salzberg C et al 2014 | Plyometric training: effectiveness and optimal duration for children with unilateral cerebral palsy. |
| 11 | Su IYW et al 2013 | Treadmill training with partial body weight support compared with conventional gait training for low-functioning children and adolescents with nonspastic cerebral palsy: a two-period crossover study \ |
| 12 | Wyatt, K. et al 2011 | Cranial osteopathy for children with cerebral palsy: a randomised controlled trial. |
| **Intervention or control does not meet inclusion criteria (n=3)** | | |
| 1 | Casey R et al 1987 | Minor head trauma in children: an intervention to decrease functional morbidity. |
| 2 | Hung WWY et al 2010 | Effects of group-based versus individual-based exercise training on motor performance in children with developmental coordination disorder: a randomized controlled study. |
| 3 | Kwon JY et al 2015 | Effect of Hippotherapy on Gross Motor Function in Children with Cerebral Palsy: a randomised controlled trial. |
| **Not RCT (n=47)** | | |
| 1 | Apache RRG 2005 | Activity-based intervention in motor skill development. Perceptual and Motor Skills |
| 2 | Baker J 1981 | A psycho-motor approach to the assessment and treatment of clumsy children. |
| 3 | Ballaz L et al 2011 | Group aquatic training improves gait efficiency in adolescents with cerebral palsy. |
| 4 | Ballaz L et al 2012 | Effect of forced use therapy on posture in children with hemiplegic cerebral palsy: a pilot study |
| 5 | Beckung E 2014 | Making it possible - interventions for children with cerebral palsy. |
| 6 | Bertoti DB 1988 | Effect of therapeutic horseback riding on posture in children with cerebral palsy. |
| 7 | Bower E et al 1996 | A randomised controlled trial of different intensities of physiotherapy and different goal-setting procedures in 44 children with cerebral palsy. |
| 8 | Buckon C et al 204 | Comparison of three ankle-foot orthosis configurations for children with spastic diplegia. |
| 9 | Casady RL et al 2004 | The effect of hippotherapy on ten children with cerebral palsy |
| 10 | Cole KN et al 1989 | Comparison of two service delivery models: in-class and out-of-class therapy approaches |
| 11 | Green D et al 2008 | Does subtype of developmental coordination disorder count: is there a differential effect on outcome following intervention? |
| 12 | Ferguson GD et al 2013 | The efficacy of two task-orientated interventions for children with Developmental Coordination Disorder: Neuromotor Task Training and Nintendo Wii Fit Training. |
| 13 | Hamilton M et al 1999 | Parent-Assisted Instruction in a Motor Skill Program for At-Risk Preschool children. |
| 14 | Humphries TW et 1992 | A comparison of the effectiveness of sensory integrative4 therapy and perceptual motor training in treating children with learning disabilities. |
| 15 | Jarus T et al 1995 | The effect of kinesthetic stimulation on the acquisition and retention of a gross motor skill by children with and without sensory integration disorders. |
| 16 | Jelsma D et al 2014 | The impact of Wii Fit intervention on dynamic balance control in children with probable Developmental Coordination Disorder and balance problems. |
| 17 | Jenkins JR et al 1982 | Effects of developmental therapy on motor-impaired children. |
| 18 | Jonsson-Funk M not RCT | Preschool motor skills following physical and occupational therapy services among non-disabled very low birth weight children. |
| 19 | Kaplan BJ et al 1993 | Reexamination of sensory integration treatment: A combination of 2 efficacy studies. |
| 20 | Kwon J-Y et al 2011 | Effects of hippotherapy on gait parameters in children with bilateral spastic cerebral palsy. |
| 21 | Luna-Oliva L et al 2013 | Kinect Xbox 360 as a therapeutic modality for children with cerebral palsy in a school environment: A preliminary study. |
| 22 | [Meyer-Heim A](http://www-ncbi-nlm-nih-gov.ezproxy2.library.usyd.edu.au/pubmed/?term=Meyer-Heim%20A%5BAuthor%5D&cauthor=true&cauthor_uid=25051931) et al 2014 | Robot-assisted and computer-based neurorehabilitation for children: the story behind |
| 23 | MacPhail AH et al 1998 | Trunk postural reactions in children with and without cerebral palsy during therapeutic horseback riding. |
| 24 | McCoy SW et al 2014 | Development and validity of the early clinical assessment of balance for young children with cerebral palsy. |
| 25 | McGibbon NH et al 1998 | Effect of an equine-movement program on gait, energy expenditure,and motor function in children with spastic cerebral palsy: a pilot study |
| 26 | Morton J et al 2005 | The effects of progressive resistancetraining for children with cerebral palsy. |
| 27 | Niemeijer AS et al 2003 | Verbal actions of physiotherapists to enhance motor learning in children with DCD. |
| 28 | Neijmeijer AS et al 2006 | Are teaching principals associated with improved motor performance in children with developmental co-ordination disorder? |
| 29 | Niemeijer AS et al 2007 | Neuromotor task training for children with developmental coordination disorder: a controlled trial |
| 30 | Olama KA et al 2011 | Endurance exercises versus treadmill training in improving muscle strength and functional activities in hemiparetic cerebral palsy. |
| 31 | Pless M et al 2000 | Effects of group motor skill intervention on 5- to 6-year-old children with developmental coordination disorder. |
| 32 | Provost B et al 2007 | Endurance and gait in children with cerebral palsy after intensive body weight-supported treadmill training. |
| 33 | Schoemaker MM et al 1994 | Physiotherapy for clumsy children - An evaluation study. |
| 34 | Schoemaker M et al 2003 | Effectiveness of neuromotor task training for children with developmental coordination disorder: A pilot study. |
| 35 | Sellick KJ et al 1980 | Effects of vestibular stimulation on motor development of cerebral palsied children. |
| 36 | Sims K et al 1996 | The remediation of clumsiness I: an evaluation of Laszlo’s kinaesthetic approach |
| 37 | Steinbok P et al 1997 | A randomised clinical trial to compare selective posterior rhizotomy plus physiotherapy with physiotherapy alone in children with spastic diplegia cerebral palsy. |
| 38 | Sugden D et al 2007 | Stability and change in children with developmental coordination disorder. |
| 39 | [Sukal-Moulton T](http://www-ncbi-nlm-nih-gov.ezproxy2.library.usyd.edu.au/pubmed/?term=Sukal-Moulton%20T%5BAuthor%5D&cauthor=true&cauthor_uid=24792141) et al 2014 | Clinical application of a robotic ankle training program for cerebral palsy compared to the research laboratory application: does it translate to practice? |
| 40 | Thorpe DE et al 2005 | The effects of an aquatic resistive exercise program on ambulatory children with cerebral palsy. |
| 41 | Unger M et al 2013 | Effect of a trunk-targeted intervention using vibration on posture and gait in children with spastic type cerebral palsy: a randomized control trial. |
| 42 | Wang X et al 2008 | Therapeutic effects on spastic cerebral palsy in children: acupuncture and massage at the Shu and He acupoints versus routine acupoints |
| 43 | Watemberg N et al 2007 | Developmental coordination disorder in children with attention-deficit-hyperactivity disorder and physical therapy intervention. |
| 44 | Watkins S et al 2014 | Preschool motor skills following physical and occupational therapy services among non-disabled very low birth weight children. |
| 45 | Westendorp M et al 2014 | Effect of a ball skill intervention on children's ball skills and cognitive functions. |
| 46 | Wright T et al 1973 | Physiotherapy forthe spastic child: an evaluation. |
| 47 | Wright FV 2014 | Measuring advanced motor skills in children with cerebral palsy: further development of the Challenge module. |
| **Data not provided at first cross over point in cross-over randomised controlled trial (n=2)** | | |
| 1 | Ho C-L et al 2006 | Functional electrical stimulation changes dynamic resources in children with spastic cerebral palsy. |
| 2 | Straker L et al 2015 | A cross-over randomised and controlled trial on the impact of active video games on motor coordination and perceptions of physical ability in children at risk of Developmental Coordination Disorder |
| **Participants have poorly defined conditions (n=8)** | | |
| 1 | Chia CLC et al 2002 | Effects of physiotherapy on school-aged children with developmental coordination disorder and learning difficulties: A pilot study. |
| 2 | de Gangi GA et al 1993 | A comparison of structured sensorimotor therapy and child-centered activity in the treatment of preschool children with sensorimotor problems. |
| 3 | Hamilton M et al 1999 | Parent-Assisted Instruction in a Motor Skill Program for At-Risk Preschool. |
| 4 | Humphries TW et al 1993 | Clinical Evaluation of the Effectiveness of Sensory Integrative and Perceptual Motor Therapy in Improving Sensory Integrative Function in Children With Learning Disabilities |
| 5 | Polatajko HJ 1991 | The effect of a sensory integration program on academic achievement, motor performance, and self-esteem in children identified as learning disabled: results of a clinical trial. |
| 6 | Salem Y et al 2012 | Effectiveness of a low-cost virtual reality system for children with developmental delay: a preliminary randomised single-blind controlled trial. |
| 7 | Watter P et al 1987 | Patterns of Improvement in Neurological Functioning of Children with Minimal Cerebral Dysfunction with Physiotherapy Intervention. |
| 8 | Winchester P et al 2002 | The effect of therapeutic horseback riding on gross motor function and gait speed in children who are developmentally delayed. |
| **Not published in English (n=8)** | | |
| 1 | Dimitrijevic L et al 2012 | Dimitrijevic L, Bjelakovic B, Lazovic M, Stankovic I, Colovic H, Kocic M, et al. [Aquatic exercise in the treatment of children with cerebral palsy]. [Serbian]. |
| 2 | Kandrali I et al 2006 | The influence of an adapted exercise program on the development of the gross motor function and performance in adolescents with spastic hemiplegia [Greek]. |
| 3 | Leemrijse C et al 2001 | De effectiviteit van Le Bon Depart en Sensorische Integratie voor kinderen met Developmental Coordination Disorder. [Dutch] |
| 4 | Jiang Q et al 2006 | The effect of functional strength training in spastic cerebral plasy [Chinese] |
| 5 | Stark C et al 2013 | Interval-rehabilitation and home-based training for children with cerebral palsy. [German] |
| 6 | Yang L et al 2009 | Effect of conductive education combined with Frenkel training on balance disability in children with cerebral palsy [Chinese]. |
| 7 | Yildirim SB et al 2012 | Yildirim Sik B, Cekmece C, Dursun N, Dursun E, Balikci E, Altunkanat Z, et al. Is hyppotherapy beneficial for rehabilitation of children with cerebral palsy? [Turkey] |
| 8 | Zhao L 2001 | Clinical controlled study of acupuncture treating children with cerebral palsy [Chinese]. |
| **No Gross Motor Outcome measure (n=15)** | | |
| 1 | Bertrand J 2009 | Interventions for children with fetal alcohol spectrum disorders (FASDs): Overview of findings for five innovative research projects. |
| 2 | Brandao MB et al 2013 | Comparison of structured skill and unstructured practice during intensive bimanual training in children with unilateral spastic cerebral palsy. |
| 3 | Casey R et al 1987 | Minor head trauma in children: an intervention to decrease functional morbidity. |
| 4 | Demuth SK et al 2012 | The PEDALS stationary cycling intervention and health-related quality of life in children with cerebral palsy: a randomized controlled trial. |
| 5 | Dodd KJ et al 2004 | Strength training can have unexpected effects on the self-concept of children with cerebral palsy. |
| 6 | Dorval G et al 1996 | Impact of aquatic programmes on adolescents with cerebral palsy. |
| 7 | Dunford C 2011 | Goal-oriented group intervention for children with developmental coordination disorder. |
| 8 | Kelly ME et al 2009 | Effects of a community-based aquatic exercise program for children with cerebral palsy: a single subject design |
| 9 | McGibbon NH et al 2009 | Immediate and long-term effects of hippotherapy on symmetry of adductor muscle activity and functional ability in children with spastic cerebral palsy. |
| 10 | Reid D et al 2006 | The use of virtual reality to improve upper extremity control in children with cerebral palsy |
| 11 | Rha DW et al 2008 | Is electrical stimulation beneficial for improving the paralytic effect of botulinum toxin type A in children with spastic diplegic cerebral palsy? |
| 12 | Salzberg C et al 2014 | Plyometric training: effectiveness and optimal duration for children with unilateral cerebral palsy. |
| 13 | Tweedy S. 1997 | Evaluation of strength and flexibility training for adolescent athletes with cerebral palsy: full report. Belconnen |
| 14 | Wilson PH et al 2002 | Motor imagery training ameliorates motor clumsiness in children. |
| 15 | Wright T et al 1973 | Physiotherapy for the spastic child: an evaluation. |
| **Not related to study age group (n=6)** | | |
| 1 | Canning CG et al 2003 | A randomized controlled trial of the effects of intensive sit-to-stand training after recent traumatic brain injury on sit-to-stand performance. |
| 2 | Cuthbert JP et al 2014 | Virtual reality-based therapy for the treatment of balance deficits in patients receiving inpatient rehabilitation for traumatic brain injury. |
| 3 | Dias D et al 2007 | Can we improve gait skills in chronic hemiplegics? A randomised control trial with gait trainer.. |
| 4 | Esquenazi A et al 2013 | A randomized comparative study of manually assisted versus robotic-assisted body weight supported treadmill training in persons with a traumatic brain injury. |
| 5 | Reddihough D 1998 | Efficacy of programmes based on conductive education for young children with CP. |
| 6 | Taub E et al 2004 | Efficacy of constraint-induced movement therapy for children with cerebral palsy with asymmetric motor impairment. |
| **Paper not retrievable (n=7)** | | |
| 1 | Wang TH et al 2012 | A randomized controlled trial of neurologic music therapy during a functional strengthening program for children with cerebral palsy. |
| 2 | Chen SW 2002 | Clinical controlled study of integrative Chinese and Western medicine in treating children with cerebral palsy. |
| 3 | Chen JJ 2002 | Clinical controlled study of Cong nao tong luo method in acupuncture treating 56 cases of children with cerebral palsy. |
| 4 | Yu CH 2008 | Clinical research on the treatment of 25 cases of children with cerebral palsy with acupuncture and tuina. |
| 5 | Zaidat A. 2007 | Effect of a motor training programme for children with cerebral palsy. |
| 6 | Zhang J 2010 | The influence of herbal bathing on the motor function in children with cerebral palsy. |
| 7 | Zhao YD 2010 | Clinical controlled study of Warming-promotion method of acupuncture treating 30 cases children with cerebral palsy |
